# Red Anthocyanins and Yellow Carotenoids Form the Color of Orange-Flower Gentian (*Gentiana lutea* L. var. *aurantiaca*)

**DOI:** 10.1371/journal.pone.0162410

**Published:** 2016-09-02

**Authors:** Judit Berman, Yanmin Sheng, Lourdes Gómez Gómez, Tania Veiga, Xiuzhen Ni, Gemma Farré, Teresa Capell, Javier Guitián, Pablo Guitián, Gerhard Sandmann, Paul Christou, Changfu Zhu

**Affiliations:** 1 Departament de Producció Vegetal i Ciència Forestal, Universitat de Lleida-Agrotecnio Center, Lleida, Spain; 2 School of Life Sciences, Changchun Normal University, Changchun, China; 3 Instituto Botánico, Departamento de Ciencia y Tecnología Agroforestal y Genética, Facultad de Farmacia, Universidad de Castilla-La Mancha, Albacete, Spain; 4 Departamento de Botánica, Universidad de Santiago de Compostela, Galicia, Spain; 5 Biosynthesis Group, Molecular Biosciences, Goethe University Frankfurt, Frankfurt, Germany; 6 ICREA, Catalan Institute for Research and Advanced Studies, Barcelona, Spain; Zhejiang University, CHINA

## Abstract

Flower color is an important characteristic that determines the commercial value of ornamental plants. Gentian flowers occur in a limited range of colors because this species is not widely cultivated as a cut flower. *Gentiana lutea* L. var. *aurantiaca* (abbr, *aurantiaca*) is characterized by its orange flowers, but the specific pigments responsible for this coloration are unknown. We therefore investigated the carotenoid and flavonoid composition of petals during flower development in the orange-flowered gentian variety of *aurantiaca* and the yellow-flowered variety of *G*. *lutea* L. var. *lutea* (abbr, *lutea*). We observed minor varietal differences in the concentration of carotenoids at the early and final stages, but only *aurantiaca* petals accumulated pelargonidin glycosides, whereas these compounds were not found in *lutea* petals. We cloned and sequenced the anthocyanin biosynthetic gene fragments from petals, and analyzed the expression of these genes in the petals of both varieties to determine the molecular mechanisms responsible for the differences in petal color. Comparisons of deduced amino acid sequences encoded by the isolated anthocyanin cDNA fragments indicated that chalcone synthase (CHS), chalcone isomerase (CHI), anthocyanidin synthase 1 (ANS1) and ANS2 are identical in both *aurantiaca* and *lutea* varieties whereas minor amino acid differences of the deduced flavonone 3-hydroxylase (F3H) and dihydroflavonol 4-reductase (DFR) between both varieties were observed. The *aurantiaca* petals expressed substantially higher levels of transcripts representing *CHS*, *F3H*, *DFR*, *ANS* and UDP-glucose:flavonoid-3-*O*-glucosyltransferase genes, compared to *lutea* petals. Pelargonidin glycoside synthesis in *aurantiaca* petals therefore appears to reflect the higher steady-state levels of pelargonidin synthesis transcripts. Moreover, possible changes in the substrate specificity of DFR enzymes may represent additional mechanisms for producing red pelargonidin glycosides in petals of *aurantiaca*. Our report describing the exclusive accumulation of pelargonidin glycosides in *aurantiaca* petals may facilitate the modification of gentian flower color by the production of red anthocyanins.

## Introduction

Flower pigmentation reflects the accumulation of secondary metabolites such as anthocyanins, carotenoids and betalains within petal epidermal cells [1]. Carotenoids and anthocyanins are the major pigments in petals, and are associated with the yellow/orange and red/blue color ranges, respectively [2,3]. *Gentiana*, a genus of plants in the family Gentianaceae, comprises more than 400 species that grow mainly in the mountainous areas of temperate regions in Asia, Europe, and the Americas, as well as Australia and New Zealand [4]. Some gentian species are used as ornamental plants, including Japanese gentians (*Gentiana scabra* and *G*. *triflora* and their hybrids) which are among the most popular floricultural plants in Japan [5]. The blue flowers of *G*. *triflora* (hereafter abbreviated to *triflora*) predominantly reflect the accumulation of gentiodelphin, a unique polyacylated delphinidin-type anthocyanin [6]. The pink flowers of *G*. *scabra* accumulate cyanidin derivatives (gentiocyanins) rather than delphinidins [7,8]. Pink-flowered gentian plants were bred from spontaneous mutations of blue-flowered varieties, reflecting the insertion of transposable elements in the flavonoid 3',5'-hydroxylase (F3'5'H) gene [8]. White-flowered gentians (*G*. *triflora* cv. Homoi and *G*. *triflora* x *G*. *scabra* cv. Polano White) do not accumulate anthocyanins in their petals due to the functional deficiency of the enzyme anthocyanidin synthase (ANS) or the transcription factor MYB3, which regulates the *ANS* gene [8,9]. The characteristic yellow petal color of *G*. *lutea* reflects the accumulation of carotenoids such as β-carotene, antheraxanthin, α-cryptoxanthin, β-cryptoxanthin, neoxanthin, violaxanthin, and predominantly lutein [10,11]. *G*. *lutea* is a medicinal plant rich in pharmacologically relevant groups of compounds, such as iridoids, secoiridoids and xanthones [12].

The Cantabrian Mountains extend more than 300 km in an east-west direction across northern Spain, from the Basque Country to Galicia. *G*. *lutea* typically produces yellow corollas, but at the southwestern tip of its distribution range (Iberian Peninsula), the species blossoms with orange flowers. Across the Cantabrian Mountains, *G*. *lutea* flower color varies longitudinally, with orange flowers (*G*. *lutea* L. var. *aurantiaca*, hereafter abbreviated to *aurantiaca*) to the west, yellow flowers (*G*. *lutea* L. var. *lutea*, hereafter abbreviated to *lutea*) to the east and both present in the transition zone [13]. The pigments that contribute to the orange petal color of *aurantiaca* flowers are unknown [14–16].

We analyzed the petals of *aurantiaca* and *lutea* flowers to determine the carotenoid and flavonoid contents. We also isolated and compared the key anthocyanin biosynthetic gene fragments (cDNAs) and their deduced amino acid sequences, and compared the expression levels of these genes involved in the anthocyanin pathway to determine the molecular mechanism responsible for the differences in petal color. Our data provide important insights into the molecular basis of pigmentation in *aurantiaca* flowers, which may facilitate the modification of gentian flower color by the production of red anthocyanins.

## Materials and Methods

### Plant materials

**Fig 1:**
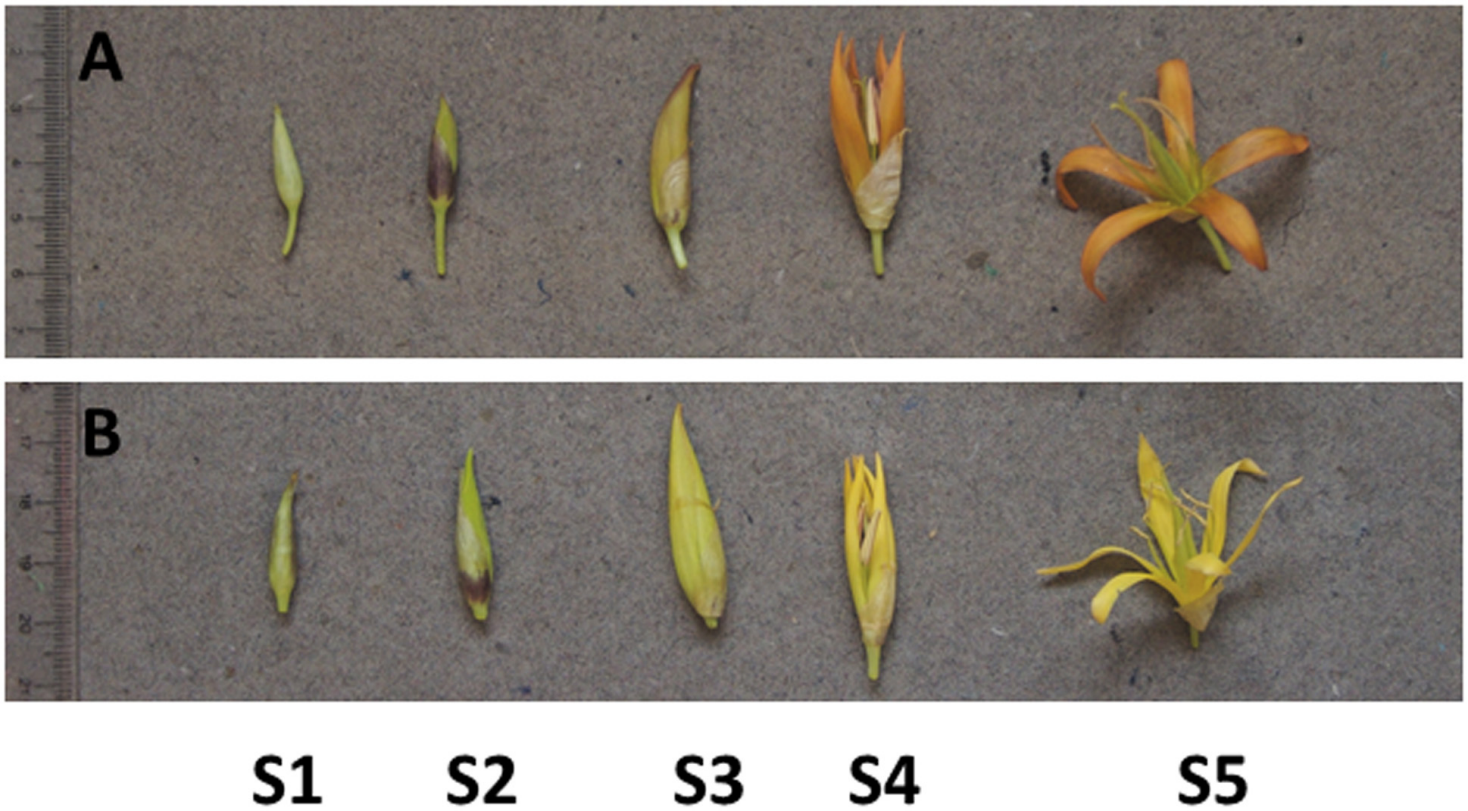
Stages of flower development in gentian varieties of *aurantiaca* (A) and *lutea* (B). Stage 1 (S1), bud less than 1.5 cm in length; stage 2 (S2), bud 1.5–2.5 cm in length; stage 3 (S3), bud 2.5–3.5 cm in length with petals slightly loosened and pigment becoming evident; stage 4 (S4), bud >3.5 cm in length with pigmentation and petals fully loosened; stage 5 (S5), fully mature, open and pigmented flower. Abbreviations: *aurantiaca*, *G*. *lutea* L. var. *aurantiaca*; *lutea*, *G*. *lutea* L. var. *lutea*.

*Gentiana lutea* L. var. *aurantiaca* (orange flowers) and *Gentiana lutea* L. var. *lutea* (yellow flowers) varieties (Fig 1) were collected from Torrestío, León (Spain) in the Eurosiberian phytogeographic region (43° 03' N; 6° 04' W; 1600 m above sea level). Petal tissue was collected at five stages of floral development and freeze dried. Each developmental stage was defined in terms of flower bud length as follows: stage 1, *<*1.5 cm long with hard bud; stage 2, 1.5–2.5 cm long; stage 3, 2.5–3.5 cm long with petals slightly loosened and the first pigmentation; stage 4, *>*3.5 cm long with colored buds and petals fully loosened; and stage 5, fully mature open and pigmented flower. All samples were stored at –80°C. For access to public land, the Territorial Service of Government of León issued permission following the relevant laws (Art. 36 and 37.1, Law 8/1991, 10 of May; Art 72.1 of the P.O.R.N. of Parque Regional de Picos de Europa en Castilla y León; Art 45.4, Law 42/2007, 13 of December).

### Extraction and quantification of total carotenoids

Carotenoids were extracted from freeze-dried petals by heating in methanol containing 6% KOH for 20 min at 60°C. The extract was partitioned into 10% ether in petroleum ether (boiling point 40–60°C), the upper phase was collected and the solvent was evaporated to dryness. After re-dissolving the residue in acetone, the carotenoids were analyzed by high performance liquid chromatography (HPLC) on a 15-cm Nucleosil C18, 3μ column with a mobile phase of acetonitrile/methanol/2-propanol (85:10:5) at 32°C. Peaks of absorbance at 450 nm were recorded using a Kontron DAD 440 photodiode array detector (Kontron Instruments, Neufahrn, Germany) and were identified by comparison with authentic standards together with their absorbance spectra. Carotenoid standards were generated by heterologous expression of appropriate genes in *E*. *coli* [17].

### Isolation and characterization of anthocyanins

The freeze-dried petals were reduced to powder under liquid nitrogen, extracted with methanol/acetic acid/water (10:1:9) by maceration with a pestle followed by vortexing, and then centrifuged at 10,000 rpm for 10 min. The supernatant was collected for HPLC analysis on a Konik HPLC system (Barcelona, Spain) equipped with a Sugerlabor Inertsil ODS 2.5-μm C18 column (250 × 4.6 mm) and connected on line to a photodiode array detector, with a range from ultraviolet to visible (200–700 nm). The column was developed with solvent system B (90% acetonitrile and 0.1% trifluoroacetic acid) and A (10% acetonitrile and 0.1% trifluoroacetic acid) under the following conditions: 0–20 min 95% A and 5% B; 20–21 min 35% A and 65% B; 21–23 min 10% A and 90% B; 23–33 min 100% methanol, and then 10 min under the initial conditions at a flow rate of 0.8 mL/min. Compounds were characterized by their elution time and absorption at 500 nm.

### Acidic hydrolysis of anthocyanins

Anthocyanin aglycones were detected by treating the powdered petal tissue with 3.6 N HCl in the dark at 100°C for 90 min, then extracting the hydrolysis mixture with isoamyl alcohol to remove the anthocyanidins for direct analysis by HPLC as described above. Anthocyanidins were identified by comparison with the standards cyanidin, delphinidin, malvidin, petunidin (TransMIT, Marburg, Germany) and pelargonidin (Sigma).

### Nucleic acid isolation and cDNA synthesis

Total RNA was isolated using the RNeasy Plant Mini Kit (Qiagen, Valencia, California, USA) and DNA was removed with DNase I (RNase-free DNase Set, Qiagen). The integrity of RNA was confirmed by gel electrophoresis in a denaturing 1.2% (w/v) agarose gel containing formaldehyde. Total RNA was quantified using a Nanodrop 1000 spectrophotometer (Thermo Scientific, Vernon Hills, Illinois, USA), and 1 μg total RNA was used as template for first strand cDNA synthesis with QuantiTect^®^ Reverse Transcription Kit (Qiagen) in a 20 μl total reaction volume, following the manufacturer’s recommendations.

### Cloning and sequencing the anthocyanin biosynthetic gene fragments from petals

A 2 μl aliquot of the first strand cDNA product was used in a 50 μl RT-PCR reaction containing 1x Green GoTaq^®^ Reaction Buffer (Promega, Madison, Wisconsin, USA), 0.2 mM of each dNTP, 0.5 μM of each primer, and 1.25 U GoTaq^®^ DNA Polymerase (Promega). Reverse transcription polymerase chain reaction (RT-PCR) primer sequences (S1 Table) designed based on the anthocyanin gene sequences from *triflora* were used to amplify partial protein coding sequences of cDNAs for chalcone synthase (*CHS*), chalcone isomerase (*CHI*), flavonone 3-hydroxylase (*F3H*), dihydroflavonol 4-reductase (*DFR*), anthocyanidin synthase (*ANS*), UDP-glucose:flavonoid-3-*O*-glucosyltransferase (*3GT*), flavonoid 3'-hydroxlase (*F3´H*) and flavonoid 3',5'-hydroxylase (*F3´5´H*) genes. The PCR conditions for all the genes involved heating the reaction to 95°C for 3 min followed by 35 cycles at 95°C for 45 s, 60°C for 45 s and 72°C for 90 s, and a final extension step at 72°C for 10 min. The resulting expected size products were purified from a 1.0% w/v agarose gel using the Geneclean^®^ II Kit (BIO^®^ 101 Systems, Solon, OH, USA) and cloned in pGEM^®^-T easy vector (Promega, Madison, WI, USA) for sequencing using the Big Dye Terminator v3.1 Cycle Sequencing Kit on a 3130x1 Genetic Analyzer (Applied Biosystems, Foster City, CA, USA). At least 20 clones were sequenced for single copy genes for *CHS*, *CHI*, *3GT*, *F3´H* and *F3´5´H*, and more than 50 clones were sequenced for two copy genes for *F3H*, *DFR* and *ANS* till more than three independent clones give the identical sequences for each isolated gene fragment. The nucleotide sequences were translated to the respective deduced amino acid sequences using Vector NTI^®^ software which was also used for alignments of cDNA and deduced amino acid sequences, respectively. All cloned genes showed sequence similarity to previously characterized anthocyanin structural genes, and BLAST searches against the National Center for Biotechnology Information (NCBI) nucleotide and protein databases were used to confirm homology with previously characterized anthocyanin genes.

### qRT-PCR analysis of anthocyanin structural gene expression in petals

From the aboved cloned cDNA sequences, gene-specific primers were designed for each of the anthocyanin genes for expression analysis by quantitative real-time RT-PCR (qRT-PCR) (S2 Table). Specific primers that amplified a single fragment of no more than 200 bp were designed in regions of the coding sequences that were isolated from orange-flowered *aurantiaca* petals. The specific primers are identical for both orange-flowered *aurantiaca* and yellow-flowered *lutea* varieties for *CHS*, *CHI*, *F3H*, *DFR* and *ANS*, and also identical for two different *F3H* (*F3H1 and F3H2*), *DFR* (*DFR1* and *DFR2*) and *ANS* (*ANS1* and *ANS2*) which permit detection of two isoform expression for *F3H*, *DFR* and *ANS*, respectively. qRT-PCR was carried out using a BioRad CFX96^™^ system. Each 25 μl reaction comprised 5 ng of cDNA, 1x iQ SYBR green supermix (BioRad, Hercules, CA, USA), and 5 μM of the each forward and reverse primers. Relative expression levels were calculated on the basis of serial dilutions of cDNA (100–0.16 ng) which were used to generate standard curves for each gene. Triplicate amplifications (biological replicates) were carried out in 96-well optical reaction plates by first heating to 95°C for 5 min followed by 40 cycles at 95°C for 30 s, 58°C for 30 s and 72°C for 30 s. Amplification specificity was confirmed by product melt curve analysis over the temperature range 50–90°C with fluorescence acquired after every 0.5°C increase. The fluorescence threshold value and gene expression data were calculated with BioRad CFX96^™^ software. Data were calculated from three biological replicates with at least three technical replicates for each and with error bars representing the standard deviation. Amplification efficiencies were compared by plotting the ΔCt values of different primer combinations of serial dilutions against the log of starting template concentrations using the CFX96^™^ software. The Ct values were adjusted to the standard curves and were normalized against the levels of ubiquitin (*UBQ*) gene.

## Results

### Carotenoid profiles during gentian flower development

**Fig 2:**
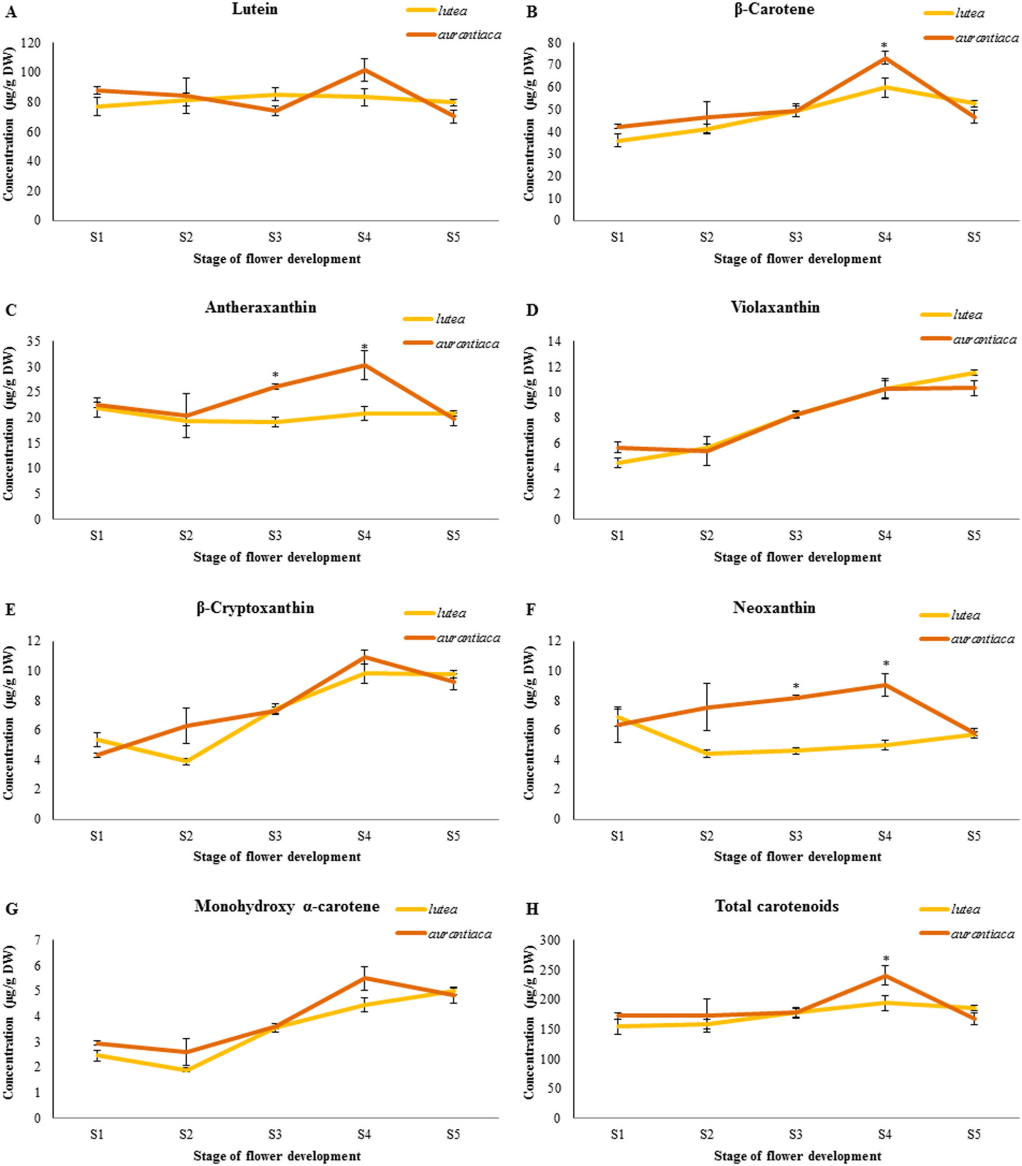
Changes in carotenoid composition profiles and total carotenoid levels in petals of *aurantiaca* and *lutea*. Abbreviations: *aurantiaca*, *G*. *lutea* L. var. *aurantiaca*; *lutea*, *G*. *lutea* L. var. *lutea*; S1, stage 1; S2, stage 2; S3, stage 3; S4, stage 4 (S4); S5, stage 5. The standard bars were obtained from three determinations from three different petal batches, and the results were given as mean ± SD. Sterisk shows very significant difference with a *P* < 0.01 (statistical analysis: T-test).

The carotenoid profiles of orange (*aurantiaca*) and yellow (*lutea*) gentian flowers (Fig 1) were analyzed by HPLC through five stages of development and similar compositions were observed in both varieties. The most abundant carotenoid in both cases was lutein (42–51% of the total), followed by β-carotene (23–31%) and antheraxanthin (11–15%), and then the minor components monohydroxy α-carotenes, β-cryptoxanthin, violaxanthin and neoxanthin (S3 and S4 Tables). The developmental profiles of the individual and total carotenoids in both varieties are shown in Fig 2. The total carotenoid content of the petals was slightly higher in *aurantiaca* than *lutea* at the early stages (S1 to S2), and the levels were similar at stage S3, *aurantiaca* again had a higher content at stage S4, but by stage S5 there was a higher overall carotenoid content in *lutea* petals (Fig 2). Nevertheless, there were no significant differences between the varieties at the early stages (S1, S2 and S3) or in the final stage (S5) except at S4 there was significant differences between orange (*aurantiaca*) and yellow (*lutea*) gentian petals. At stage S3, antheraxanthin was more abundant in *aurantiaca* than *lutea*. Lutein, β-carotene and antheraxanthin were more abundant in *aurantiaca* than *lutea* at stage S4. Furthermore, neoxanthin was more abundant in *aurantiaca* than *lutea* at stages S2, S3 and S4, while neoxanthin was at the similar levels at stages S1 and S5. The monohydroxy α-carotenes, β-cryptoxanthin and violaxanthin levels in the petals were similar in both varieties throughout development.

### Flavonoid profiles during gentian flower development

**Fig 3:**
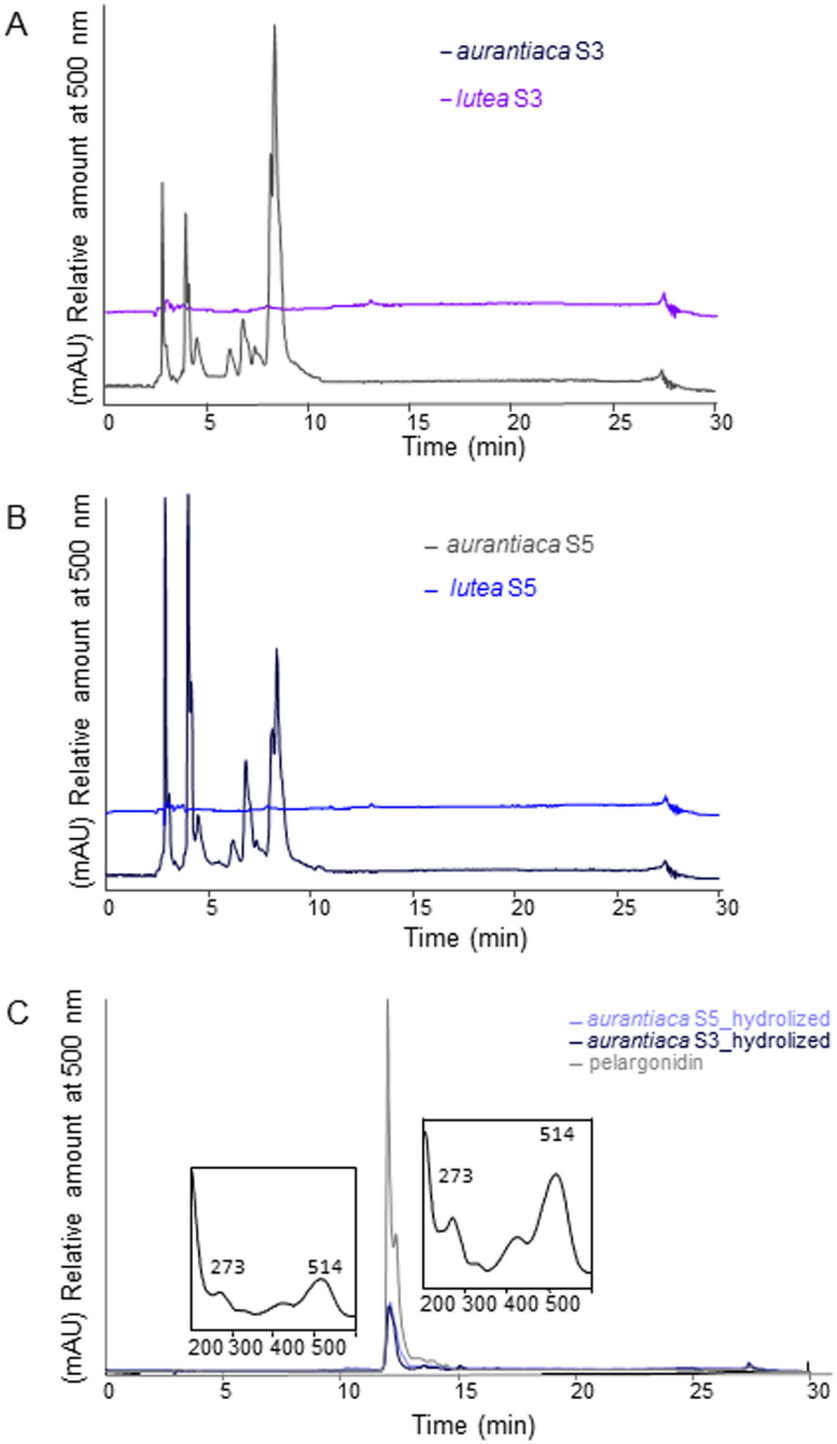
Analysis of anthocyanin profiles in the petals of *aurantiaca* and *lutea*. Chromatograms of (**A**) crude extracts from the yellow petal variety *lutea* and orange petal variety *aurantiaca* in the developmental stage S3 (**B**) crude extracts from the yellow petal variety *lutea* and orange petal variety *aurantiaca* in the developmental stage S5. (**C**) Hydrolyzed extracts from petals at two developmental stages (S3 and S5) representing the orange petal variety *aurantiaca*. Absorbance at 500 nm was used for the detection of anthocyanins. The chromatogram of the anthocyanin standard pelargonidin is shown in gray. Insets showing the UV-visible spectra of the hydrolysates (left) and pelargonidin (right). Abbreviations: *aurantiaca*, *G*. *lutea* L. var. *aurantiaca*; *lutea*, *G*. *lutea* L. var. *lutea*; S3, stage 3; S5, stage 5.

As above, there were no significant differences in the total carotenoid content of the petals between orange (*aurantiaca*) and yellow (*lutea*) varieties at stages S3 and at the final stage S5. Thus, the flavonoid profiles of orange (*aurantiaca*) and yellow (*lutea*) gentian flowers were analyzed by HPLC at stages S3 and S5 of flower development (Fig 3 and S1–S3 Figs). The yellow *lutea* petals were completely devoid of anthocyanin pigments at stages S3 and S5 whereas anthocyanins were present in *aurantiaca* petals at the corresponding stages (Fig 3A and 3B). HPLC analysis of the hydrolyzed anthocyanin extracts from *aurantiaca* petals revealed the exclusive accumulation of the anthocyanidin pelargonidin (Fig 3C). The extracts were also analyzed for the presence of other phenolic pigments such as flavones and flavonols, but these were detected at very low levels (S3 Fig). The relative quantitation of compounds with an absorbance spectrum in the range 200–400 nm revealed no major differences between the *aurantiaca* and *lutea* varieties (S1A Fig). In contrast, we observed clear differences in the relative quantities of pelargonidin glycosides, which have an absorbance peak at 500 nm (S1B Fig).

### Cloning of anthocyanin structural genes

**Fig 4:**
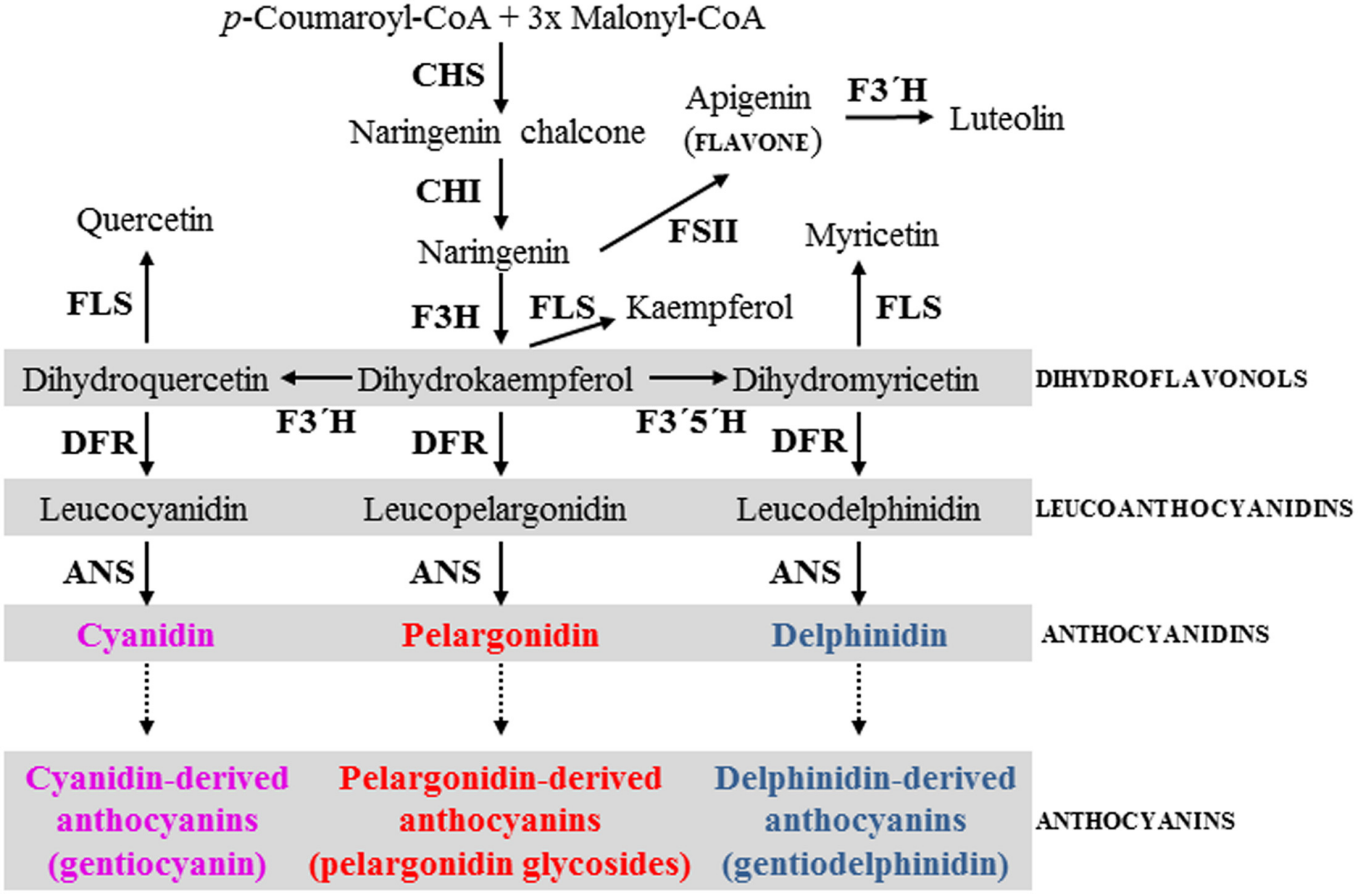
The gentian flavonoid biosynthesis pathway. Flavonol synthase (FLS) is not required for pigment production, but is necessary for the synthesis of the three flavonols: kaempferol, quercetin and myricetin. Anthocyanidins (pelargonidin, cyanidin, and delphinidin) are modified by the addition of sugars and other moieties to form anthocyanins in a species-specific manner. Pink-flowered gentians accumulate gentiocyanin anthocyanin in their petals, and blue-flowered gentians accumulate mainly gentiodelphin anthocyanin, whereas orange-flowered gentian accumulates exclusively pelargonidin glycosides in the petals. Abbreviations: ANS, anthocyanidin synthase; CHI, chalcone isomerase; CHS, chalcone synthase; DFR, dihydroflavonol 4-reductase; F3H, flavanone 3-hydroxylase; F3'H, flavonoid 3'-hydroxlase; F3'5'H, flavonoid 3',5'-hydroxlase; FLS, flavonol synthase; FSII, flavone synthase.

**Fig 5:**
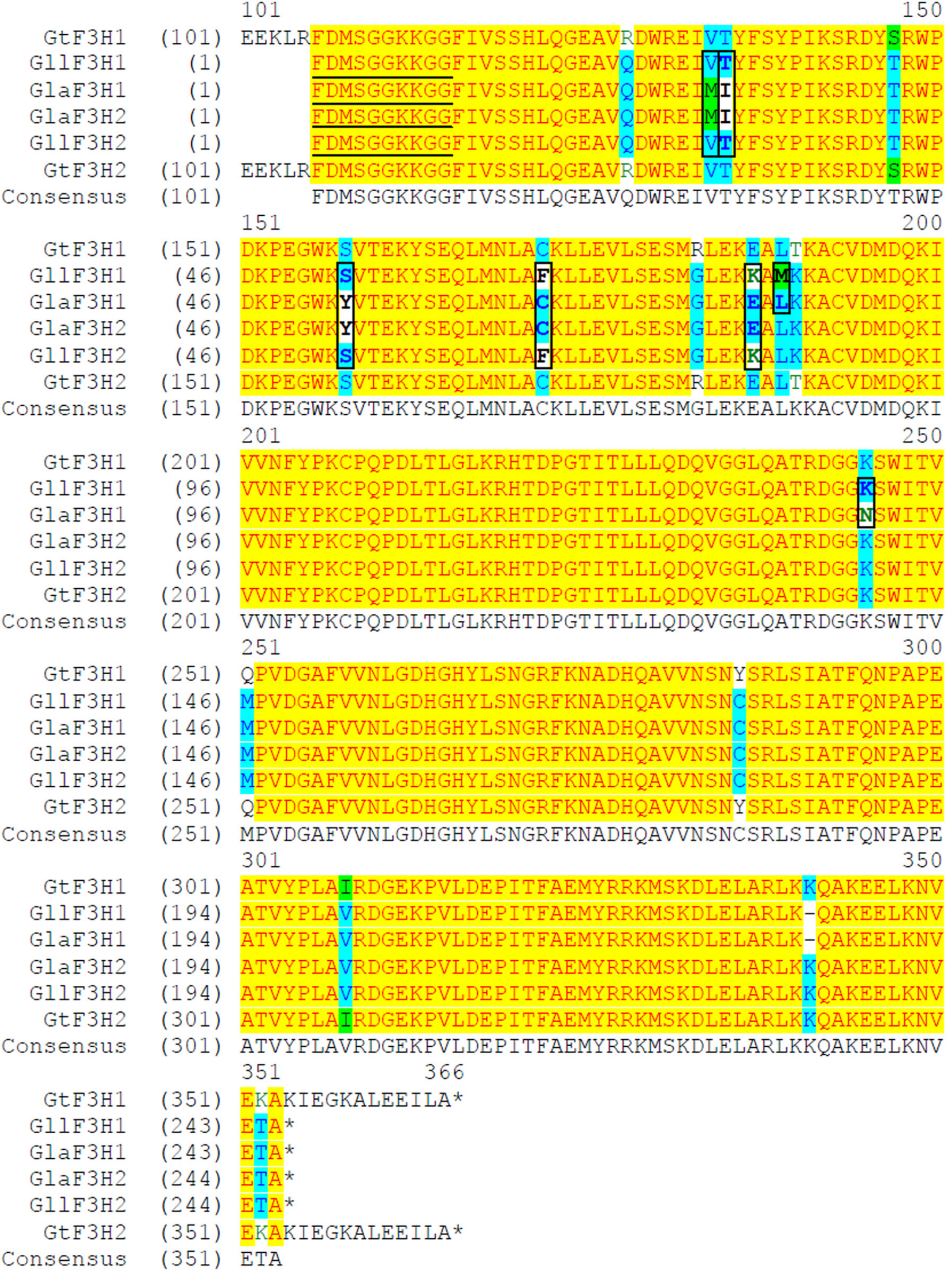
Multiple alignments of the deduced amino acid sequences encoded by flavonone 3-hydroxylase (*F3H*) cDNAs. The stop codon is marked with the asterisk. The different deduced amino acids of *F3H* orthologues between *lutea* and *aurantiaca* varieties are marked with square shape. The underlined amino acid sequences from *lutea* and *aurantiaca* were deduced from primers. Gaps are insered with a dash (-) in one of the sequences. Abbreviations: *aurantiaca*, *G*. *lutea* L. var. *aurantiaca*; *lutea*, *G*. *lutea* L. var. *lutea*; Gt, *G*. *triflora*; Gll, *G*. *lutea* L. var. *lutea*; Gla, *G*. *lutea* L. var. *aurantiaca*; F3H, flavonone 3-hydroxylase. GenBank accession numbers: GtF3H1, AB193311; GtF3H2, AB193312. The *F3H* cDNAs from *lutea* and *aurantiaca* are isolated by authors in this study.

**Fig 6:**
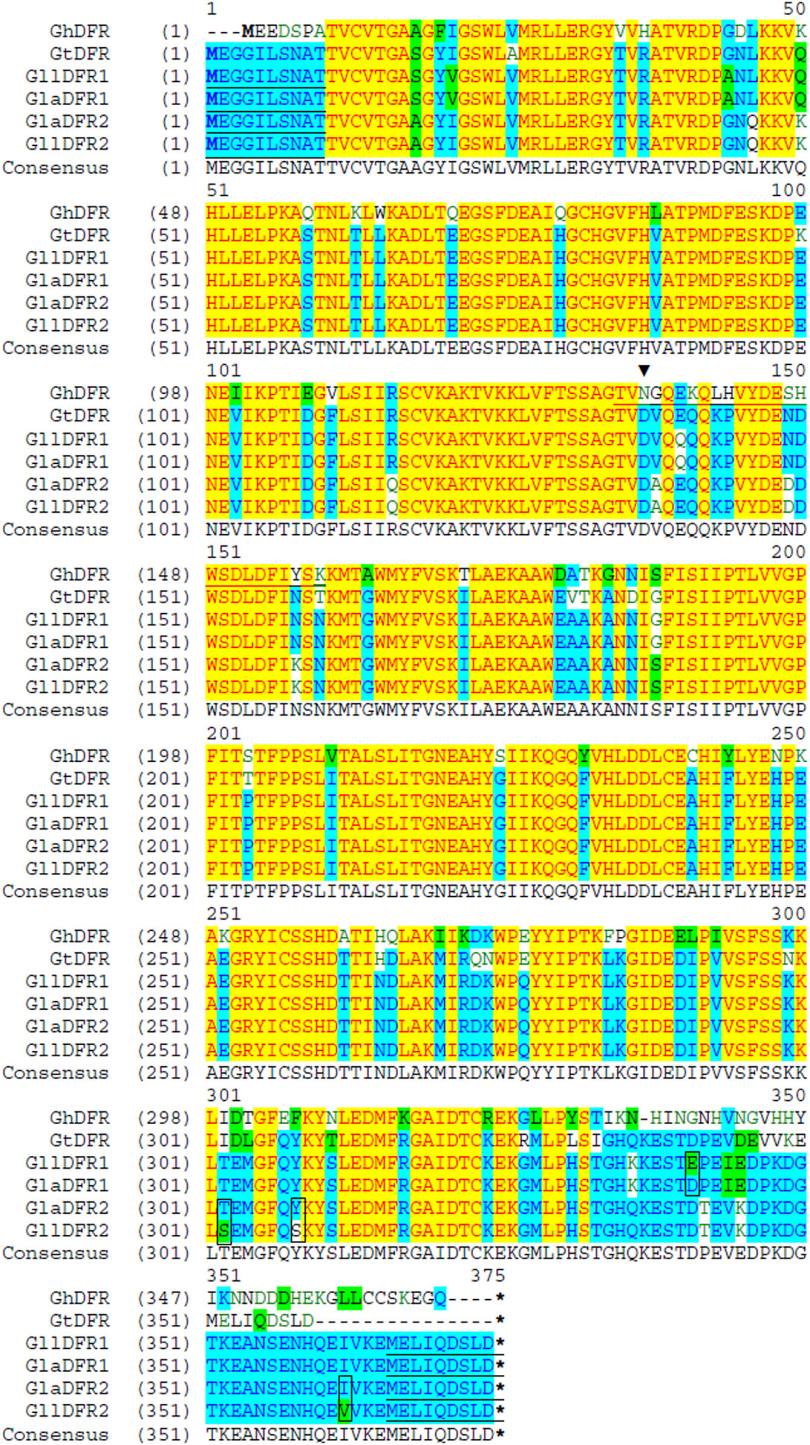
Multiple alignments of the deduced amino acid sequences encoded by dihydroflavonol 4-reductase (*DFR*) cDNAs. The first methionine (M) and and stop codon are marked with bold letter and the asterisk, respectively. The 134th asparagine (N) for *Gerbera hybrida* DFR is marked by an arrowhead. The underlined amino acid sequences from *G*. *hybrida* indicate the substrate specificity determining region identified by Johnson et al. [26]. The different deduced amino acids of *DFR* orthologues between *lutea* and *aurantiaca* are marked with square shape. Gaps are insered with a dash (-) in one of the sequences. The underlined amino acid sequences from *lutea* and *aurantiaca* were deduced from primers. Abbreviations: *aurantiaca*, *G*. *lutea* L. var. *aurantiaca*; *lutea*, *G*. *lutea* L. var. *lutea*; Gh, *Gerbera hybrida*; Gt, *G*. *triflora*; Gll, *G*. *lutea* L. var. *lutea*; Gla, *G*. *lutea* L. var. *aurantiaca*; DFR, dihydroflavonol 4-reductase. GenBank accession numbers: GhDFR, CAA78930; GtDFR, D85185. The *DFR* cDNAs from *lutea* and *aurantiaca* are isolated by authors in this study.

At least five different enzymatic reactions catalyzed by CHS, CHI, F3H, DFR and ANS (Fig 4) must occur to produce red pelargonidin pigment, and the genes encoding for these enzymes have been characterized in several model species [18, 19]. Genes encoding the gentian enzymes responsible for anthocyanin biosynthesis from *G*. *triflora* [20–23] provide sequences that can be used for the design of primers to clone the orthologous genes from *G*. *lutea* L. var. *lutea* and *G*. *lutea* L. var. *aurantiaca* varieties. We therefore produced a panel of primers (S1 Table) to clone anthocyanin gene fragments from mixed petals of stages S3 and S5 by RT-PCR. Each pair of gene primers amplified a single product of the anticipated size. Cloning, sequencing and alignments of these cDNA fragments and their deduced encoding proteins (Figs 5 and 6; S4 and S5 Figs) demonstrated all the isolated anthocyanin cDNAs from *aurantiaca* and *lutea* varieties had high identity (more than 88.0%, eliminating primer sequences) with those from *triflora*. Alignments of the orthologous cDNA sequences (eliminating primer sequences) between *lutea* and *aurantiaca* varieties showed 100% identity for *CHS* and *CHI*, and more than 95.0% identity for *F3H*, *DFR* and *ANS* (S4 Fig). Although one cDNA for both *DFR* and *ANS* genes was cloned from petals of blue-flowered *triflora* [20,23], two different cDNAs for both *DFR* and *ANS* genes were isolated from the petals of the orange-flowered *aurantiaca* and yellow-flowered *lutea* varieties (S4 Fig). The absence of pelargonidin glycosides from the petals of yellow-flowered *lutea* variety indicates that the pelargonidin pathway is blocked in *lutea* petals. Inspection of all the cloned cDNA sequences did not identify any obvious substitutions that introduced a frameshift or premature stop codon that would suggest nonfunctional enzymes in the yellow-flowered *lutea* variety. Anthocyanidins are converted to the anthocyanidin 3-glucoside by UDP-glucose:flavonoid-3-*O*-glucosyltransferase (3GT), which is particularly well conserved in flowering plants in the glycosyltransferase [19,24,25]. Two related P450s, hydroxylases of flavonoid 3'-hydroxlase (F3´H) and flavonoid 3',5'-hydroxylase (F3´5´H) convert dihydrokaempferol into dihydroquercetin and dihydromyricetin, respectively (Fig 4). The *3GT*, *F3´H* and *F3´5´H* cDNA fragments (S4 Fig) were also isolated from petals of the orange-flowered *aurantiaca* variety in order to design specific primers for gene expression analysis.

Comparisons of deduced amino acid sequences encoded by the isolated anthocyanin cDNAs indicated that CHS, CHI, ANS1 and ANS2 are identical in both *aurantiaca* and *lutea* varieties (S5 Fig). F3H1 and F3H2 sequences shared 99.2% amino acid sequence identity in both varieties (Fig 5). Between *aurantiaca* and *lutea* varieties, the sequence identities for F3H1 and F3H2 were 97.0% and 97.9% respectively. There are seven and five amino acid differences for F3H1 and F3H2 respectively, between *aurantiaca* and *lutea* varieties (Fig 5), which occurred in the middle regions of F3H. DFR1 and DFR2 showed 94.6% and 95.8% amino acid sequence identities in *lutea* and *aurantiaca* varieties, respectively (Fig 6). The sequence identities for DFR1 and DFR2 were 99.7% and 99.2% respectively between *aurantiaca* and *lutea* varieties. DFR1 copies between *aurantiaca* and *lutea* varieties differ by one amino acid, while DFR2 copies between *aurantiaca* and *lutea* varieties differ by three amino acids (Fig 6). These differences are located in the regions near the C-terminus.

### Anthocyanin structural gene expression

**Fig 7:**
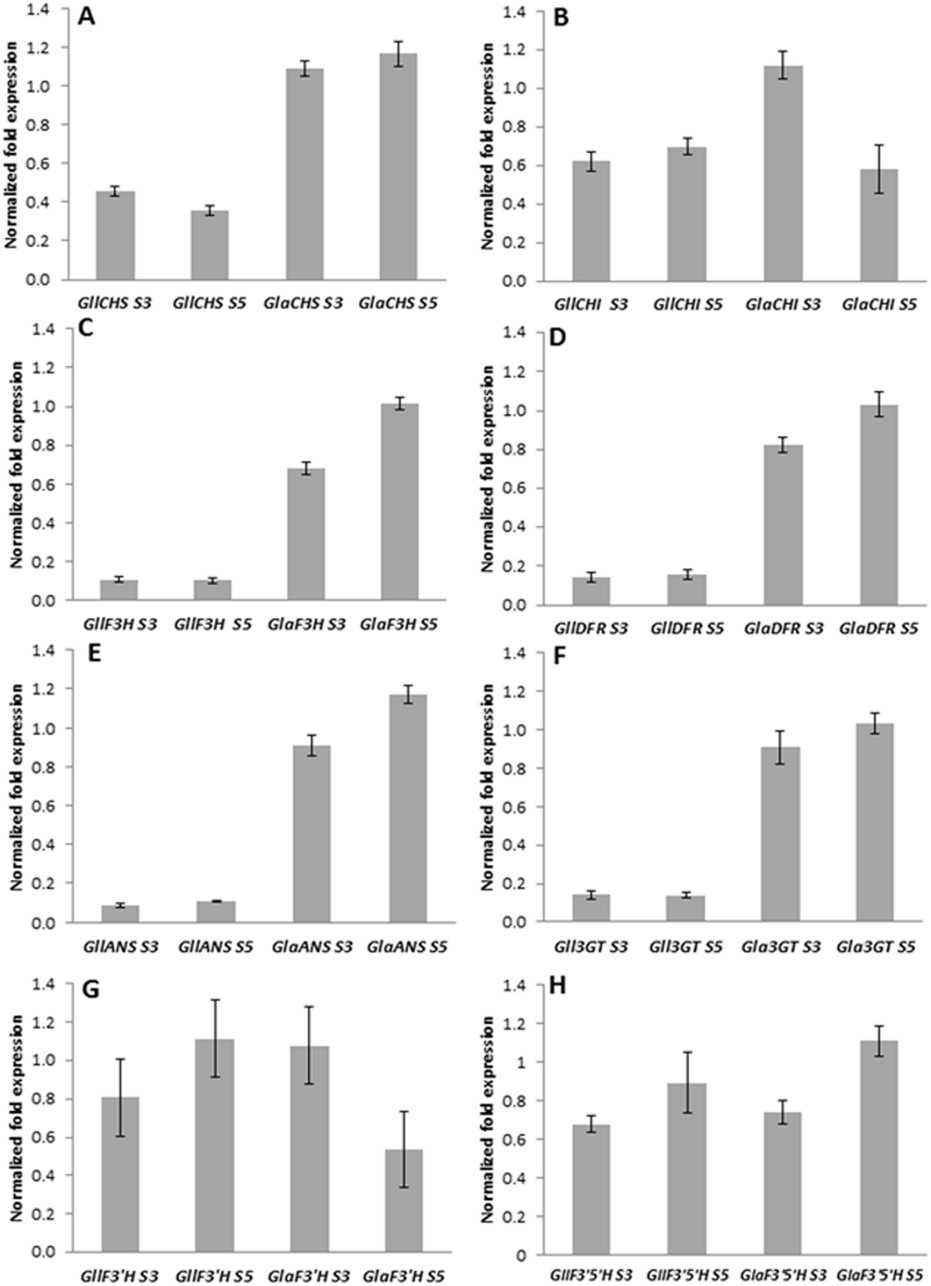
Quantitative expression of anthocyanin genes normalized to ubiquitin gene in petals of *lutea* and *aurantiaca*. Abbreviations: *aurantiaca*, *G*. *lutea* L. var. *aurantiaca*; *lutea*, *G*. *lutea* L. var. *lutea*; Gll, *G*. *lutea* L. var. *lutea*; Gla, *G*. *lutea* L. var. *aurantiaca*; CHS, chalcone synthase; CHI, chalcone isomerase; F3H, flavonone 3-hydroxylase; DFR, dihydroflavonol 4-reductase; ANS, anthocyanidin synthase; 3GT, UDP-glucose:flavonoid-3-*O*-glucosyltransferase; F3'H, flavonoid 3'-hydroxlase; F3'5'H, flavonoid 3',5'-hydroxylase. Data were calculated from three biological replicates with at least three technical replicates for each and with error bars representing the standard deviation.

The genes coding for the enzymes of the anthocyanin pathway typically regulated at the levels of transcription [19,27]. We compared expression levels of the anthocyanin biosynthetic genes from petal tissues from the orange-flowered *aurantiaca*, and the yellow-flowered *lutea* using quantitative RT-PCR (qRT-PCR) to determine whether variation in anthocyanin gene expression could account for differential activities of the pelargonidin branch of the pathway in the orange-flowered *aurantiaca*, and the yellow-flowered *lutea*. Quantitative assays using qRT-PCR demonstrated that the *aurantiaca* petals contained substantially higher levels of chalcone synthase (*CHS*), flavonone 3-hydroxylase (*F3H*), dihydroflavonol 4-reductase (*DFR*), anthocyanidin synthase (*ANS*), and UDP-glucose:flavonoid-3-*O*-glucosyltransferase (*3GT*) mRNA than the *lutea* petals at both stages (S3 and S5) (Fig 7). The *aurantiaca* petals accumulated higher relative levels of chalcone isomerase (*CHI*) mRNA at stage S3 than the *lutea* petals, while the similar levels of chalcone isomerase (*CHI*) mRNA at stage S5 were observed between *aurantiaca* and *lutea* petals (Fig 7B). The similar levels of flavonoid 3'-hydroxlase (*F3'H*) mRNA accumulated in both the *lutea* and *aurantiaca* petals at stage S3, while higher relative levels of flavonoid 3'-hydroxlase (*F3'H*) mRNA was observed in *lutea* petals than *aurantiaca* petals at stage S5 (Fig 7G). The petals of both varieties of *aurantiaca* and *lutea* accumulated the similar levels of flavonoid 3',5'-hydroxylase (*F3'5'H*) mRNA at both stages (S3 and S5) (Fig 7H).

## Discussion

Flower color variation in gentian is limited compared to other colored plant species such as chrysanthemum, rose and carnation, which may explain why gentian is not widely cultivated as a cut flower [5]. The genus *Gentiana* comprises more than 400 species, including *G*. *triflora* with blue flowers resulting from the accumulation of minor cyanidin and major delphinidin derivatives (602 μg/g fresh weight delphinidin) in petals [6,8,9], and *G*. *scabra* with pink flowers with exclusive accumulation of gentiocyanins (cyanidin derivatives) anthocyanins in petals [7,8], both of which have similar amounts of gentiocyanins but do not accumulate carotenoids in petals [8]. *G*. *triflora* and *G*. *scabra* are important ornamental plants and common hybridization parents in Japan [5], as well as white-flowered gentians (*G*. *triflora* cv. Homoi and *G*. *triflora* x *G*. *scabra* cv. Polano White) which do not accumulate anthocyanins and carotenoids in their petals [8,9]. In addition to the blue, pink and white flowers of the species described above, *G*. *lutea* is a species of gentian that usually has yellow petals, reflecting the accumulation of lutein and other carotenoids (ca. 370 μg/g fresh weight total carotenoids) [10,11]. However, the flower color varies across the Cantabrian Mountains, with orange flowers predominant in the west (*G*. *lutea* L. var. *aurantiaca*) and yellow flowers predominant in the east (*G*. *lutea* L. var. *lutea*). The metabolic basis of the orange flower has not been characterized thus far [14–16].

Three alternative ways are known to generate orange petal pigments in plants–the accumulation of more total carotenoids, the specific accumulation of more red-pigment carotenoids, or the accumulation of red-pigment anthocyanins. Orange pigmentation achieved by increasing total carotenoids is common among the Compositae [28]. Orange petals are formed in this way in the African marigold (*Tagetes erecta*) [29], French marigold (*T*. *petula*) [28] and sunflower (*Helianthus annuus*) [28]. Orange and yellow varieties of these species show only minor variations in the anthocyanin and carotenoid profiles but the total carotenoid content of the orange petals is higher than that of yellow petals. In other species, orange petal color is achieved by accumulating more carotenoids representing the red end of the pigmentation spectrum, e.g. capsanthin, rubixanthin and lycopene. Orange petals are formed in this way in the California poppy (*Eschscholzia californica*) [30], gazania (*Gazania* spp.) [28] and African daisy (*Osteospermum ecklonis*) [28]. The third way, the accumulation of more anthocyanins, is used to achieve the orange petal color of chrysanthemum (*Chrysanthemum morifolium*), gerbera (*Gerbera jamesonii*) and zinnia (*Zinnia elegans*). Yellow and orange varieties of these species show little difference in carotenoid levels or profiles, but the orange petals contain higher levels of anthocyanins [28].

In order to determine which of these ways confers the orange petal color in the gentian variety *aurantiaca*, we analyzed total carotenoids and carotenoid profiles in the *aurantiaca* and *lutea* petals. We observed only minor differences between the varieties in terms of total carotenoid content at the early stages (S1,S2 and S3) or in the final stage (S5) except at S4 (Fig 2H; S3 and S4 Tables) and found that the red-spectrum carotenoids were entirely absent, effectively ruling out both carotenoid-dependent ways for the formation of orange petals. Therefore, it seems likely that the petal color is determined by a combination of carotenoid and anthocyanin pigments, with the latter playing a major role in creating the orange pigmentation in the *aurantiaca* variety. These results directed our investigation toward flavonoid accumulation, and cloning and expression of genes involved in flavonoid biosynthesis in the *aurantiaca* and *lutea* petals.

We sought to confirm the above hypothesis by analyzing flavonoid levels in the petals of each variety. This duly revealed the presence of pelargonidin glycosides (brick-red color) [18] in the *aurantiaca* petals but the complete absence of this compound in the *lutea* petals (Fig 3). We found no obvious differences among the yellow, and orange petals in the levels of other flavonoids at stages S3 and S5 (S1–S3 Figs) and total carotenoids (Fig 2H). Therefore, we can report for the first time that the characteristic orange petal color of the gentian *G*. *lutea* L. var. *aurantiaca* distributed at the western half of the Cantabrian Range is caused by a combination of anthocyanin and carotenoid pigments, with pelargonidin glycosides playing a key role in color determination.

The differences in anthocyanin accumulation in petals between *aurantiaca* and *lutea* varieties are determined by the transcriptional regulation of genes encoding the enzymes responsible for anthocyanin pathway. Most of the pelargonidin-derived anthocyanin pathway genes (*CHS*, *F3H*, *DFR*, *ANS* and 3*GT*) were expressed at substantially higher levels in *aurantiaca* petals than *lutea* petals (Fig 7). In contrast, as compared to the *lutea* petals, the *aurantiaca* petals expressed similar or lower levels of *F3'H* and *F3'5'H* mRNA, encoding enzymes involved in cyanidin and delphinidin biosynthesis, respectively (Figs 4, 7G and 7H). These results suggest that the presence versus absence of pelargonidin-derived anthocyanin pigments in the orange-flowered *aurantiaca* and yellow-flowered *lutea* petals may be predominantly due to the expression differences of pelargonidin-derived anthocyanin pathway genes, but they do not rule out the possibility that DFR enzymes from the yellow-flowered *lutea* petals are nonfunctional since there are minor differences in the deduced amino acid sequences encoded by the isolated *DFR* cDNAs between *aurantiaca* and *lutea* varieties (Fig 6). Alternatively, DFR enzymes from the yellow-flowered *lutea* petals might be the inability to catalyze dihydrokaempferol reduction (Fig 4). Although there are minor differences between *aurantiaca* and *lutea* varieties in the deduced amino acid sequences encoded by the isolated *F3H* gene fragments (Fig 5), the F3H1 and/or F3H2 enzyme(s) must be functional in *lutea* variety due to the accumulation of low levels of flavonols in petals.

The exclusive accumulation of pelargonidin glycosides in petals of orange-flowered *aurantiaca* could be achieved by alteration of the substrate specificity of dihydroflavonol 4-reductase (DFR) enzymes. DFR converts dihydroflavonoids (dihydrokaempferol, dihydroquercetin, and dihydromyricetin) into leucoanthocyanidins, which are subsequently modified to form three colored anthocyanidins (pelargonidin, cyanidin, and delphinidin) by anthocyanidin synthase (ANS) (Fig 4). DFR competes with flavonol synthase (FLS) for dihydroflavonols as common substrates and therefore interferes with flavonol formation [31]. The substrate specificity of DFR often helps to determine which anthocyanin compounds accumulate. In some plant species, such as petunia (*Petunia hybrida*) and cymbidium (*Cymbidium hybrida*), DFR has strict substrate specificity and cannot utilize dihydrokaempferol (DHK) efficiently [32,33]. This is why these species lack pelargonidin-based anthocyanins and thus lack flowers with an orange/brick red color. Blue- and pink-flowered gentian DFR enzymes may have similar substrate specificity to its petunia ortholog because pelargonidin-based anthocyanins are not found in these gentian plants [20]. The blue and pink Japanese gentian varieties do not contain pelargonidin-based anthocyanins as major floral pigments, because pelargonidin is only found at residual levels in the petals of blue-flowered *G*. *triflora* [23]. However, in some genera, like gerbera (*Gerbera hybrida*), DFR is able to efficiently reduce all three substrates [26]. The exchange of the 134th asparagine (N) into a non-polar leucine (L) converts the non-specific gerbera DFR into a DFR that preferentially reduces DHK (Fig 6) [26], implying the 134th asparagine in the gerbera DFR plays an important role in substrate specificity. The 134th asparagine is conserved in most DFRs that are known to accept DHK as a substrate while petunia DFR has aspartic acid [26]. The presence of an aspartic acid in the petunia DFR sequence was suggested to determine the inability of converting DHK [26]. In the DFRs from strawberry (*Fragagaria* x *ananasa* cv. Elsanta), amino acid in 133 (corresponding to gerbera DFR amino acid 134) is an asparagine in DFR2 but is an alanine in DFR1 [34]. The recombinant DFR1 from strawberry prefers DHK while DFR2 converts DHQ (dihydroquercetin) and DHM (dihydromyricetin) to leucocyanindin and leucodelphinidin, but did not accept DHK as a substrate [34]. The presence of a non-polar amino acid in the strawberry DFR1 is in line with [26]. The observed substrate specificity of DFR2, however, cannot be explained by the presence of the asparagine, because this is also found in the non-specific DFR from gerbera [34]. The same as petunia, all the DFRs from blue-, yellow- and orange-flowered gentian varieties have aspartic acid (D) in this corresponding position other than asparagine (N) or non-polar amino acids (Fig 6). Moreover, the minor amino acid differences of the deduced DFRs between the orange-flowered *aurantiaca* and yellow-flowered *lutea* varieties are located in the regions near the C-terminus other than the substrate specificity determining region (amino acid 132–157 in gerbera DFR, Fig 6) identified by Johnson et al. [26]. However, since the cyanidin and delphinidin branches of the anthocyanin pathways are inactive in the petals of the orange-flowered *aurantiaca*, possible changes in the substrate specificity of DFR enzymes may represent additional mechanisms for producing red pelargonidin glycosides in petals of *aurantiaca*. It will be helpful to prove this hypothesis through investigating whether the *aurantiaca* DFR enzymes are able to preferentially reduce DHK to produce red pelargonidin in the transgenic plants (e.g.using a white flowered petunia mutant-W80 accumulating primarily DHK but with appreciate amount of DHQ, and DHM as transformation host [26]).

In addition to the structural backbone of the pathway, much is known about the regulation of the genes encoding these enzymes. In all species that have been examined, gene regulation occurs at the level of transcription *via* the interaction of *cis*-regulatory elements in the structural anthocyanin genes and functional transcription factor complexes. These regulatory complexes comprise members of the R2R3MYB, basic helix-loop-helix (bHLH), and WD40 repeat protein families [19,27,35], and activate expression of anthocyanin structural genes [19,27,36,37]. For proper transcriptional activation, the MYB protein is dependent on physical interactions with bHLH proteins, whereas the WD40 proteins are believed to serve as an anchor to support the complex [35,38]. In blue-flowered gentian (*G*. *triflora*), GtMYB3 interacts with GtbHLH1, and the complex of these two proteins activates the expression of *GtF3´5´H* and *5AT* (hydroxycinnamoyl-CoA:anthocyanin 5-*O*-acyltransferase) genes [39]. However, the GtMYB3/GtbHLH1 complex could not activate the expression of *GtCHS* gene [39]. The relatively higher co-expression of several pelargonidin-derived anthocyanin structural genes (*GlaCHS*, *GlaF3H*, *GlaDFR*, *GlaANS* and *Gla3GT*) is observed in the petals of orange-flowered *aurantiaca* as compared to yellow-flowered *lutea* (Fig 7). Provided that the pattern of coordinated regulation of anthocyanin pathway genes in orange-flowered *aurantiaca* is similar to that in blue-flowered *triflora*, if regulation of *GlaF3´5´H* in *aurantiaca* petals is caused by the orthologues GtMYB3/GtbHLH1 complex, we would not expect to see marked upregulation of *GlaCHS*, and other anthocyanin structural genes (*GlaF3H*, *GlaDFR*, *GlaANS* and *Gla3GT*), but we do (Fig 7). Thus, the independent regulation of *GlaCHS* relative to *GlaF3´5´H* suggests that changes in the *cis*-regulatory regions of the structural genes and/or in the *cis*-regulatory regions of these anthocyanin transcription factor genes, are responsible for the upregulation of these structural gene expression (*GlaCHS*, *GlaF3H*, *GlaDFR*, *GlaANS* and *Gla3GT*) in *aurantiaca* petals as compared with *lutea* petals. However, this inference remains tentative.

## Conclusions

In summary, our results show that the orange petals of *G*. *lutea* L. var. *aurantiaca* accumulate pelargonidin glycosides that are absent in the yellow petals of *G*. *lutea* L. var. *lutea*. This suggests that the orange petal color is predominantly caused by newly synthesized pelargonidin glycosides that confer a reddish hue to the yellow background color, derived primarily from the carotenoid lutein. These data provide insight into the basis of flower pigmentation in gentian plants and facilitate our understanding of the modification of gentian flower color. The ornamental gentians lack pelargonidin pigments, and therefore our investigation into the molecular regulation of anthocyanin accumulation in the petals of *G*. *lutea* L. var. *aurantiaca* and *G*. *lutea* L. var. *lutea* may facilitate the development of novel orange-flowered gentian cultivars by genetic engineering.

## Supporting Information

S1 Fig Relative amounts of flavonoids detected in the petals of *lutea* (*Gentiana lutea* L.var. *lutea*) and *aurantiaca* (*G*. *lutea* L. var. *aurantiaca*) flowers at two developmental stages (S3 and S5). **A**) Relative amounts for the most intense peaks detected between 200–400 nm range. **B**) Relative amounts for the most intense peaks detected at 500 nm. Error bars represent SD (standard deviation) values for three replicate determinations.
(DOC)

S2 Fig Characterization of phenolic compounds in the petals of gentian species. **A**) Representative HPLC-PDA/UV isoplot chromatograms showing the compounds detected (200–550nm) in petals of *lutea* (*Gentiana lutea* L.var. *lutea*) and *aurantiaca* (*G*. *lutea* L. var. *aurantiaca*) flowers at two developmental stages (S3 and S5). **B**) Absorbance spectra and retention times (in minutes) for the most intense peaks detected between 200–400 nm. **C**) Absorbance spectra and retention times (in minutes) for the most intense peaks detected at 500 nm.
(DOC)

S3 Fig Presence of flavonoids in the petals of gentian species. **A**) Representative HPLC-PDA/UV chromatogram showing the compounds detected at 370 nm in petals of *lutea* (*Gentiana lutea* L. var. *lutea*) flowers in stage S5. **B**) Representative HPLC-PDA/UV chromatogram showing the compounds detected at 370 nm in petals of *aurantiaca* (*G*. *lutea* L.var. *aurantiaca*) flowers in stage S5. **C**) Absorbance spectra and retention times (in minutes) for the most intense peaks detected at 370 nm.
(DOC)

S4 Fig Alignments of cDNA sequences encoded anthocyanin biosynthetic enzymes among *Gentiana triflora*, *G*. *lutea* L. var. *lutea* and *G*. *lutea* L. var. *aurantiaca*. The underlined cDNA sequences are primers used to isolate cDNAs from *lutea* and *aurantiaca*. The start codon (ATG) and stop codons (TGA, TAG or TAA) are underlined with bold letters. Gaps are insered with a dash (-) in one of the sequences. Abbreviations: *triflora*, *Gentiana triflora*; *aurantiaca*, *G*. *lutea* L. var. *aurantiaca*; *lutea*, *G*. *lutea* L. var. *lutea*; Gt, *Gentiana triflora*; Gll, *G*. *lutea* L. var. *lutea*; Gla, *G*. *lutea* L. var. *aurantiaca*; CHS, chalcone synthase; CHI, chalcone isomerase; F3H, flavonone 3-hydroxylase; DFR, dihydroflavonol 4-reductase; ANS, anthocyanidin synthase; 3GT, UDP-glucose:flavonoid-3-*O*-glucosyltransferase; F3´H, for flavonoid 3'-hydroxylase; F3´5´H, flavonoid 3',5'-hydroxylase. GenBank accession numbers: GtCHS, D38043; GtCHI, D38168; GtDFR, D85185; GtANS, AB193310; Gt3GT, D85186; GtF3´H, AB193313; GtF3´5´H, D85184; GtF3H1, AB193311; GtF3H2, AB193312. The cDNA sequences encoded anthocyanin biosynthetic enzymes from *lutea* and *aurantiaca* are isolated by authors in this study.
(DOC)

S5 Fig Alignments of the deduced amino acid sequences encoded by anthocyanin biosynthetic genes from *Gentiana triflora*, *G*. *lutea* L. var. *lutea*, and *G*. *lutea* L. var. *aurantiaca*. The first methionine (M) and stop codon are marked with underlined bold letter and the asterisk, respectively. Gaps are insered with a dash (-) in one of the sequences. The underlined amino acid sequences from *lutea* and *aurantiaca* were deduced from primers. Abbreviations: *triflora*, *Gentiana triflora*; *aurantiaca*, *G*. *lutea* L. var. *aurantiaca*; *lutea*, *G*. *lutea* L. var. *lutea*; Gt, *Gentiana triflora*; Gll, *G*. *lutea* L. var. *lutea*; Gla, *G*. *lutea* L. var. *aurantiaca*; CHS, chalcone synthase; CHI, chalcone isomerase; ANS, anthocyanidin synthase; 3GT, UDP-glucose:flavonoid-3-*O*-glucosyltransferase; F3´H, for flavonoid 3'-hydroxylase; F3´5´H, flavonoid 3',5'-hydroxylase. GenBank accession numbers: GtCHS, D38043; GtCHI, D38168; GtANS, AB193310; Gt3GT, D85186; GtF3´H, AB193313; GtF3´5´H, D85184; GtF3H1, AB193311; GtF3H2, AB193312. The cDNA sequences encoded anthocyanin biosynthetic enzymes from *lutea* and *aurantiaca* are isolated by authors in this study.
(DOC)

S1 Table Oligonucleotide sequences of primer pairs used for RT-PCR. Abbreviations: CHS, chalcone synthase; CHI, chalcone isomerase; F3H, flavonone 3-hydroxylase; DFR, dihydroflavonol 4-reductase; ANS, anthocyanidin synthase; 3GT, UDP-glucose:flavonoid-3-*O*-glucosyltransferase; F3'H, flavonoid 3'-hydroxlase; F3'5'H, flavonoid 3',5'-hydroxylase.
(DOC)

S2 Table Oligonucleotide sequences of primer pairs designed based on the cDNA sequences isolated from petals of *Gentiana lutea* L. var. *aurantiaca*, and ubiquitin (*UBQ*) gene [39] as reference used for quantitative real-time PCR (qRT-PCR) analysis. Abbreviations: CHS, chalcone synthase; CHI, chalcone isomerase; F3H, flavonone 3-hydroxylase; DFR, dihydroflavonol 4-reductase; ANS, anthocyanidin synthase; 3GT, UDP-glucose:flavonoid-3-*O*-glucosyltransferase; F3'H, flavonoid 3'-hydroxlase; F3'5'H, flavonoid 3',5'-hydroxylase.
(DOC)

S3 Table Concentration of carotenoid composition and content in orange petals in *Gentiana lutea* L. var. *aurantiaca* during different stages of flower development. Values are expressed as μg/g DW; Each value represents the mean result from three determinations from three different petal batches ±SD (standard deviation).
(DOC)

S4 Table Concentration of carotenoid composition and content in yellow petals of *Gentiana lutea* L. var. *lutea* during different stages of flower development. Each value represents the mean result from three determinations from three different petal batches ±SD (standard deviation).
(DOC)
