# Supplementary material for: Red Anthocyanins and Yellow Carotenoids Form the Color of Orange-Flower Gentian (Gentiana lutea L. var. aurantiaca)
Source: PLoS One. 2016 Sep 2;11(9):e0162410. doi: 10.1371/journal.pone.0162410 (PMC5010251; doi:10.1371/journal.pone.0162410)
Supplement: S1 Fig — A) Relative amounts for the most intense peaks detected between 200–400 nm range. B) Relative amounts for the most intense peaks detected at 500 nm. Error bars represent SD (standard deviation) values for three replicate determinations. (DOC) [file pone.0162410.s001.doc]

**S1 Fig. Relative amounts of flavonoids detected in the petals of *lutea* (*Gentiana lutea* L*.*var. *lutea*) and *aurantiaca* (*G. lutea* L. var. *aurantiaca*) flowers at two developmental stages (S3 and S5)**. **A**) Relative amounts for the most intense peaks detected between 200-400 nm range. **B**) Relative amounts for the most intense peaks detected at 500 nm. Error bars represent SD (standard deviation) values for three replicate determinations.
