# Supplementary material for: Red Anthocyanins and Yellow Carotenoids Form the Color of Orange-Flower Gentian (Gentiana lutea L. var. aurantiaca)
Source: PLoS One. 2016 Sep 2;11(9):e0162410. doi: 10.1371/journal.pone.0162410 (PMC5010251; doi:10.1371/journal.pone.0162410)
Supplement: S2 Fig — A) Representative HPLC-PDA/UV isoplot chromatograms showing the compounds detected (200–550nm) in petals of lutea (Gentiana lutea L.var. lutea) and aurantiaca (G. lutea L. var. aurantiaca) flowers at two developmental stages (S3 and S5). B) Absorbance spectra and retention times (in minutes) for the most intense peaks detected between 200–400 nm. C) Absorbance spectra and retention times (in minutes) for the most intense peaks detected at 500 nm. (DOC) [file pone.0162410.s002.doc]

**S2** **Fig.** **Characterization of phenolic compounds in the petals of gentian species.** **A**) Representative HPLC-PDA/UV isoplot chromatograms showing the compounds detected (200-550nm) in petals of *lutea* (*Gentiana lutea* L*.*var. *lutea*) and *aurantiaca* (*G. lutea* L*.* var. *aurantiaca*) flowers at two developmental stages (S3 and S5). **B**) Absorbance spectra and retention times (in minutes) for the most intense peaks detected between 200-400 nm. **C**) Absorbance spectra and retention times (in minutes) for the most intense peaks detected at 500 nm.
