# Supplementary material for: Red Anthocyanins and Yellow Carotenoids Form the Color of Orange-Flower Gentian (Gentiana lutea L. var. aurantiaca)
Source: PLoS One. 2016 Sep 2;11(9):e0162410. doi: 10.1371/journal.pone.0162410 (PMC5010251; doi:10.1371/journal.pone.0162410)
Supplement: S3 Fig — A) Representative HPLC-PDA/UV chromatogram showing the compounds detected at 370 nm in petals of lutea (Gentiana lutea L. var. lutea) flowers in stage S5. B) Representative HPLC-PDA/UV chromatogram showing the compounds detected at 370 nm in petals of aurantiaca (G. lutea L.var. aurantiaca) flowers in stage S5. C) Absorbance spectra and retention times (in minutes) for the most intense peaks detected at 370 nm. (DOC) [file pone.0162410.s003.doc]

**S3 Fig. Presence of flavonoids in the petals of gentian species.** **A**) Representative HPLC-PDA/UV chromatogram showing the compounds detected at 370 nm in petals of *lutea* (*Gentiana lutea* L*.* var. *lutea*) flowers in stage S5. **B**) Representative HPLC-PDA/UV chromatogram showing the compounds detected at 370 nm in petals of *aurantiaca* (*G. lutea* L*.*var. *aurantiaca*) flowers in stage S5. **C**) Absorbance spectra and retention times (in minutes) for the most intense peaks detected at 370 nm.
