# Supplementary material for: Red Anthocyanins and Yellow Carotenoids Form the Color of Orange-Flower Gentian (Gentiana lutea L. var. aurantiaca)
Source: PLoS One. 2016 Sep 2;11(9):e0162410. doi: 10.1371/journal.pone.0162410 (PMC5010251; doi:10.1371/journal.pone.0162410)
Supplement: S4 Fig — The underlined cDNA sequences are primers used to isolate cDNAs from lutea and aurantiaca. The start codon (ATG) and stop codons (TGA, TAG or TAA) are underlined with bold letters. Gaps are insered with a dash (-) in one of the sequences. Abbreviations: triflora, Gentiana triflora; aurantiaca, G. lutea L. var. aurantiaca; lutea, G. lutea L. var. lutea; Gt, Gentiana triflora; Gll, G. lutea L. var. lutea; Gla, G. lutea L. var. aurantiaca; CHS, chalcone synthase; CHI, chalcone isomerase; F3H, flavonone 3-hydroxylase; DFR, dihydroflavonol 4-reductase; ANS, anthocyanidin synthase; 3GT, UDP-glucose:flavonoid-3-O-glucosyltransferase; F3´H, for flavonoid 3'-hydroxylase; F3´5´H, flavonoid 3',5'-hydroxylase. GenBank accession numbers: GtCHS, D38043; GtCHI, D38168; GtDFR, D85185; GtANS, AB193310; Gt3GT, D85186; GtF3´H, AB193313; GtF3´5´H, D85184; GtF3H1, AB193311; GtF3H2, AB193312. The cDNA sequences encoded anthocyanin biosynthetic enzymes from lutea and aurantiaca are isolated by authors in this study. (DOC) [file pone.0162410.s004.doc]

**A**

51 100

GtCHS (51) TTTTTCCGGCGATCAAGA**ATG**GTGACCGTTGAGGAGATCAGAAAAGCTCA

GllCHS (1) GATCAAGA**ATG**GTGACCGTTGAGGAGATCAGAAATGCTCA

GlaCHS (1) GATCAAGA**ATG**GTGACCGTTGAGGAGATCAGAAATGCTCA

Consensus (51) GATCAAGAATGGTGACCGTTGAGGAGATCAGAAATGCTCA

101 150

GtCHS (101) GAGAGCCGAAGGTCCAGCCACCGTCTTGGCTATCGGCACCGCTACGCCGG

GllCHS (41) GAGAGCCGAAGGTCCAGCCACCGTCTTGGCAATCGGCACCGCTACACCGA

GlaCHS (41) GAGAGCCGAAGGTCCAGCCACCGTCTTGGCAATCGGCACCGCTACACCGA

Consensus (101) GAGAGCCGAAGGTCCAGCCACCGTCTTGGCAATCGGCACCGCTACACCGA

151 200

GtCHS (151) TTAATTGTGTGGATCAGAGCACTTATCCGGATTATTACTTCCGGATCACT

GllCHS (91) TTAATTGTGTGGACCAAAGCACTTATCCGGATTATTATTTCCGGATCACT

GlaCHS (91) TTAATTGTGTGGACCAAAGCACTTATCCGGATTATTATTTCCGGATCACT

Consensus (151) TTAATTGTGTGGACCAAAGCACTTATCCGGATTATTATTTCCGGATCACT

201 250

GtCHS (201) GATAGCGAGCATAAGACTGAGCTGAAAGAGAAATTCAAGCGCATGTGTGA

GllCHS (141) GACAGCGAGCATAAGACTGAGCTCAAAGAGAAATTCAAGCGCATGTGTGA

GlaCHS (141) GACAGCGAGCATAAGACTGAGCTCAAAGAGAAATTCAAGCGCATGTGTGA

Consensus (201) GACAGCGAGCATAAGACTGAGCTCAAAGAGAAATTCAAGCGCATGTGTGA

251 300

GtCHS (251) AAAATCGATGATAAGGAAAAGGTACATGCACTTGACAGAAGACATCCTAA

GllCHS (191) AAAATCAATGATAAGGCAAAGGTACATGCACTTGACAGAAGACATCCTTA

GlaCHS (191) AAAATCAATGATAAGGCAAAGGTACATGCACTTGACAGAAGACATCCTTA

Consensus (251) AAAATCAATGATAAGGCAAAGGTACATGCACTTGACAGAAGACATCCTTA

301 350

GtCHS (301) AAGAGAATCCCAACATGTGTGCATATATGGCACCTTCCCTTGATGCTAGG

GllCHS (241) AAGAAAATCCCAACATTTGTGCATATATGGCACCTTCCCTTGATGCAAGA

GlaCHS (241) AAGAAAATCCCAACATTTGTGCATATATGGCACCTTCCCTTGATGCAAGA

Consensus (301) AAGAAAATCCCAACATTTGTGCATATATGGCACCTTCCCTTGATGCAAGA

351 400

GtCHS (351) CAGGACATTGTGGTGGTTGAAGTCCCAAAACTTGGGAAAGAGGCTGCCCA

GllCHS (291) CAAGACATTGTGGTGGTTGAAGTCCCAAAACTTGGTAAAGAGGCAGCCCA

GlaCHS (291) CAAGACATTGTGGTGGTTGAAGTCCCAAAACTTGGTAAAGAGGCAGCCCA

Consensus (351) CAAGACATTGTGGTGGTTGAAGTCCCAAAACTTGGTAAAGAGGCAGCCCA

401 450

GtCHS (401) GAAGGCAATTAAAGAATGGGGACAGCCCAAGTCCAAGATCACTCACCTTG

GllCHS (341) GAAGGCAATTAAAGAATGGGGCCAACCCAAGTCCAAGATCACCCACCTTG

GlaCHS (341) GAAGGCAATTAAAGAATGGGGCCAACCCAAGTCCAAGATCACCCACCTTG

Consensus (401) GAAGGCAATTAAAGAATGGGGCCAACCCAAGTCCAAGATCACCCACCTTG

451 500

GtCHS (451) TCTTTTGTACCACTAGCGGTGTGGACATGCCCGGTGCCGACTATCAGATC

GllCHS (391) TGGTTTGCACTACTAGCGGTGTGGACATGCCCGGGGCCGACTATCAAATC

GlaCHS (391) TGGTTTGCACTACTAGCGGTGTGGACATGCCCGGGGCCGACTATCAAATC

Consensus (451) TGGTTTGCACTACTAGCGGTGTGGACATGCCCGGGGCCGACTATCAAATC

501 550

GtCHS (501) ACCAAACTTCTTGGTCTGCGGTCTTCCGTGAAGCGGTTCATGATGTACCA

GllCHS (441) ACCAAACTTCTGGGCCTCCGGTCTTCCGTTAAGCGGTTCATGATGTACCA

GlaCHS (441) ACCAAACTTCTGGGCCTCCGGTCTTCCGTTAAGCGGTTCATGATGTACCA

Consensus (501) ACCAAACTTCTGGGCCTCCGGTCTTCCGTTAAGCGGTTCATGATGTACCA

551 600

GtCHS (551) ACAAGGTTGCTTTGCCGGTGGGACGGTCCTCCGTATGGCCAAGGATTTGG

GllCHS (491) ACAAGGTTGCTTTGCTGGTGGCACGGTCCTCCGTATGGCCAAGGATTTGG

GlaCHS (491) ACAAGGTTGCTTTGCTGGTGGCACGGTCCTCCGTATGGCCAAGGATTTGG

Consensus (551) ACAAGGTTGCTTTGCTGGTGGCACGGTCCTCCGTATGGCCAAGGATTTGG

601 650

GtCHS (601) CTGAGAACAACAGAGGTGCCCGCGTACTGGTAGTCTGCTCTGAGATCACC

GllCHS (541) CTGAGAACAACAGAGGTGCACGTGTTCTGGTAGTGTGCTCTGAGATCACC

GlaCHS (541) CTGAGAACAACAGAGGTGCACGTGTTCTGGTAGTGTGCTCTGAGATCACC

Consensus (601) CTGAGAACAACAGAGGTGCACGTGTTCTGGTAGTGTGCTCTGAGATCACC

651 700

GtCHS (651) GCTGTAACCTTCCGTGGACCAAGTGAGTCTCACTTGGATAGTCTTGTAGG

GllCHS (591) GCTGTAACCTTTCGTGGACCAAGTGAGTCTCACTTGGATAGTCTTGTTGG

GlaCHS (591) GCTGTAACCTTTCGTGGACCAAGTGAGTCTCACTTGGATAGTCTTGTTGG

Consensus (651) GCTGTAACCTTTCGTGGACCAAGTGAGTCTCACTTGGATAGTCTTGTTGG

701 750

GtCHS (701) ACAGGCCCTGTTTGGTGACGGCGCTGCAGCTATCATCGTGGGTTCTGATC

GllCHS (641) ACAGGCCTTGTTTGGTGACGGTGCTGCAGCTATCATCGTTGGTTCTGATC

GlaCHS (641) ACAGGCCTTGTTTGGTGACGGTGCTGCAGCTATCATCGTTGGTTCTGATC

Consensus (701) ACAGGCCTTGTTTGGTGACGGTGCTGCAGCTATCATCGTTGGTTCTGATC

751 800

GtCHS (751) CGATCCCGGACTTGGAAAGGCCGTTGTTTCAGATCGTTTCAGCAGCCCAA

GllCHS (691) CGATCCCGGACTTGGAAAGGCCGTTGTTTCAAATAGTGTCTGCAGCCCAA

GlaCHS (691) CGATCCCGGACTTGGAAAGGCCGTTGTTTCAAATAGTGTCTGCAGCCCAA

Consensus (751) CGATCCCGGACTTGGAAAGGCCGTTGTTTCAAATAGTGTCTGCAGCCCAA

801 850

GtCHS (801) ACCCTTCTTCCGGATAGCCACGGTGCCATCGACGGTCATCTTCGTGAGGT

GllCHS (741) ACACTGCTTCCCGATAGCCATGGCGCCATCGACGGGCATCTTCGTGAGGT

GlaCHS (741) ACACTGCTTCCCGATAGCCATGGCGCCATCGACGGGCATCTTCGTGAGGT

Consensus (801) ACACTGCTTCCCGATAGCCATGGCGCCATCGACGGGCATCTTCGTGAGGT

851 900

GtCHS (851) TGGGCTCACGTTTCATTTACTTAAAGACGTTCCGGGGCTTATCTCTAAGC

GllCHS (791) TGGGCTTACATTTCATCTACTTAAGGATGTTCCTGGGCTTATCTCTAAGC

GlaCHS (791) TGGGCTTACATTTCATCTACTTAAGGATGTTCCTGGGCTTATCTCTAAGC

Consensus (851) TGGGCTTACATTTCATCTACTTAAGGATGTTCCTGGGCTTATCTCTAAGC

901 950

GtCHS (901) ACATTGAGAAAAGCTTGAAAGAGGCTTTTGACCCTATCGGGATCTCTGAT

GllCHS (841) ACATTCAGAAAAGCTTGAAAGAAGCATTTGAACCCATTGGGATTTCTGAT

GlaCHS (841) ACATTCAGAAAAGCTTGAAAGAAGCATTTGAACCCATTGGGATTTCTGAT

Consensus (901) ACATTCAGAAAAGCTTGAAAGAAGCATTTGAACCCATTGGGATTTCTGAT

951 1000

GtCHS (951) TGGAACTCGATCTTCTGGATTGCACACCCTGGTGGGCCGGCTATTTTGGA

GllCHS (891) TGGAACTCCATCTTCTGGATTGCACACCCTGGTGGGCCGGCAATTCTGGA

GlaCHS (891) TGGAACTCCATCTTCTGGATTGCACACCCTGGTGGGCCGGCAATTCTGGA

Consensus (951) TGGAACTCCATCTTCTGGATTGCACACCCTGGTGGGCCGGCAATTCTGGA

1001 1050

GtCHS (1001) CCAAGTAGAGGAAACATTGGGCCTAGAAGCCCAAAAGCTGCGGGCTACGA

GllCHS (941) CCAAGTAGAGGAAACATTGGGCCTAGAAGCCCAAAAACTGCGGTCTACGA

GlaCHS (941) CCAAGTAGAGGAAACATTGGGCCTAGAAGCCCAAAAACTGCGGTCTACGA

Consensus (1001) CCAAGTAGAGGAAACATTGGGCCTAGAAGCCCAAAAACTGCGGTCTACGA

1051 1100

GtCHS (1051) GGCACGTGCTAGCTGAGTATGGGAACATGTCAAGTGCATGTGTGTTGTTT

GllCHS (991) GGCATGTTCTAAGTGAGTATGGGAACATGTCAAGTGCATGTGTGTTGTTT

GlaCHS (991) GGCATGTTCTAAGTGAGTATGGGAACATGTCAAGTGCATGTGTGTTGTTT

Consensus (1051) GGCATGTTCTAAGTGAGTATGGGAACATGTCAAGTGCATGTGTGTTGTTT

1101 1150

GtCHS (1101) ATACTAGATGAGATGAGAAAAACTTCTGCCAAAGATGGAGCAACCACTAC

GllCHS (1041) ATACTTGATGAGATGAGAAAATCTTCAGCAAGAGATGGAGCAAGCACCAC

GlaCHS (1041) ATACTTGATGAGATGAGAAAATCTTCAGCAAGAGATGGAGCAAGCACCAC

Consensus (1101) ATACTTGATGAGATGAGAAAATCTTCAGCAAGAGATGGAGCAAGCACCAC

1151 1200

GtCHS (1151) TGGAGAAGGACAAGATTGGGGTGTCCTGTTTGGGTTTGGGCCGGGCCTCA

GllCHS (1091) AGGAGAAGGCCTAGATTGGGGTGTTCTGTTTGGGTTTGGGCCAGGCCTCA

GlaCHS (1091) AGGAGAAGGCCTAGATTGGGGTGTTCTGTTTGGGTTTGGGCCAGGCCTCA

Consensus (1151) AGGAGAAGGCCTAGATTGGGGTGTTCTGTTTGGGTTTGGGCCAGGCCTCA

1201 1250

GtCHS (1201) CTGTCGAGACCGTCGTGTTACACAGCGTTTCGGTT**TGA**GAGAGCCCATCT

GllCHS (1141) CTGTCGAGACTGTCGTGTTACACAGCGTTTCGGTT**TGA**GAGAG

GlaCHS (1141) CTGTCGAGACTGTCGTGTTACACAGCGTTTCGGTT**TGA**GAGAG

Consensus (1201) CTGTCGAGACTGTCGTGTTACACAGCGTTTCGGTTTGAGAGAG

**B**

51 100

GtCHI (51) CCACATAACC**ATG**GTTTCTTCTTCAGTCTCCTCCGTCACCGAAGTTAAAG

GllCHI (1) TCCGTCACCGAAGTTAAAG

GlaCHI (1) TCCGTCACCGAAGTTAAAG

Consensus (51) TCCGTCACCGAAGTTAAAG

101 150

GtCHI (101) TCGAGAGCTACGTTTTCCCGCCGTCCGTCAAGCCTCCTTCCTCCACCAAA

GllCHI (20) TCGAGAGCTACGTTTTCCCTCCGTCCGTCAAGCCTCCTTCCTCCCCCAAA

GlaCHI (20) TCGAGAGCTACGTTTTCCCTCCGTCCGTCAAGCCTCCTTCCTCCCCCAAA

Consensus (101) TCGAGAGCTACGTTTTCCCTCCGTCCGTCAAGCCTCCTTCCTCCCCCAAA

151 200

GtCHI (151) TCCTTTCTCCTCGGCGGCGCAGGGGTGAGGGGACTGGAAATTAACGGTAA

GllCHI (70) TCCTTTCTCCTCGGCGGCGCAGGAGTGAGGGGACTGGACATTAACGGCAA

GlaCHI (70) TCCTTTCTCCTCGGCGGCGCAGGAGTGAGGGGACTGGACATTAACGGCAA

Consensus (151) TCCTTTCTCCTCGGCGGCGCAGGAGTGAGGGGACTGGACATTAACGGCAA

201 250

GtCHI (201) CTTCGTGAAGTTCACGGCGATCGGAGTTTACTTGGAAGAGAGTGGTGTTG

GllCHI (120) CTTCGTGAAGTTCACGGCGATCGGAGTTTACTTAGAAGAGAATGGTGTTG

GlaCHI (120) CTTCGTGAAGTTCACGGCGATCGGAGTTTACTTAGAAGAGAATGGTGTTG

Consensus (201) CTTCGTGAAGTTCACGGCGATCGGAGTTTACTTAGAAGAGAATGGTGTTG

251 300

GtCHI (251) CCGTACTTTCCGGCAAGTGGAAAGGCAAAACTGCTGAAGAGTTGTCGGAT

GllCHI (170) CTTTACTTGCCGGCAAGTGGAAAGGCAAAACTGCTGAAGAGTTGACCGAT

GlaCHI (170) CTTTACTTGCCGGCAAGTGGAAAGGCAAAACTGCTGAAGAGTTGACCGAT

Consensus (251) CTTTACTTGCCGGCAAGTGGAAAGGCAAAACTGCTGAAGAGTTGACCGAT

301 350

GtCHI (301) TCCGTTGAGTTCTTCACCGATATCATCACAGGTCCCTTTGAAAAGTTCAC

GllCHI (220) TCCGTTGAGTTCTTCTCCGATATCATCACAGGTCCCTTTGAAAAGCTCAC

GlaCHI (220) TCCGTTGAGTTCTTCTCCGATATCATCACAGGTCCCTTTGAAAAGCTCAC

Consensus (301) TCCGTTGAGTTCTTCTCCGATATCATCACAGGTCCCTTTGAAAAGCTCAC

351 400

GtCHI (351) TCAGGTGACACTAATCCTGCCGGTGACCGGCCAGCAATACTCTCCAAAGG

GllCHI (270) CCACGGGACATTTATCCTGCCGTTGACCGGCGAACAATACTCTGCAAAGG

GlaCHI (270) CCACGGGACATTTATCCTGCCGTTGACCGGCGAACAATACTCTGCAAAGG

Consensus (351) CCACGGGACATTTATCCTGCCGTTGACCGGCGAACAATACTCTGCAAAGG

401 450

GtCHI (401) TGGCGGAAAATTGCGCCGCCCAATGGAAAGCTGCCGGAATTTATACGGAT

GllCHI (320) TGGCGGAAAATTGCGTCGCCCAATGGAAAGCGGCCGGAATTTATACCGAT

GlaCHI (320) TGGCGGAAAATTGCGTCGCCCAATGGAAAGCGGCCGGAATTTATACCGAT

Consensus (401) TGGCGGAAAATTGCGTCGCCCAATGGAAAGCGGCCGGAATTTATACCGAT

451 500

GtCHI (451) GCAGATGGAATTGCAATTGAAAAGTTCCTTCAAGTTTTCCAAACTGAATC

GllCHI (370) GCTGACGGAATAGCAATTGAAAAATTCCTTCAAGTTTTCCAAACTAAATC

GlaCHI (370) GCTGACGGAATAGCAATTGAAAAATTCCTTCAAGTTTTCCAAACTAAATC

Consensus (451) GCTGACGGAATAGCAATTGAAAAATTCCTTCAAGTTTTCCAAACTAAATC

501 550

GtCHI (501) TTTTACTCCCGGTGATTCCATTCTTTTTACCCACTCTCCTGAATCTTTAA

GllCHI (420) TTTTACTACCGGTGACTACGTTCTTTATACCCACTCACCTGAATCTTTAA

GlaCHI (420) TTTTACTACCGGTGACTACGTTCTTTATACCCACTCACCTGAATCTTTAA

Consensus (501) TTTTACTACCGGTGACTACGTTCTTTATACCCACTCACCTGAATCTTTAA

551 600

GtCHI (551) CGATAAGTTTTGGAAAGAATGGAGCTATT---CCTGAAGTAAGCAATGCA

GllCHI (470) CGATAAGTTTTGGGAAGAATGGAGCTATTATTCCTGAAGTGGGCAATGCA

GlaCHI (470) CGATAAGTTTTGGGAAGAATGGAGCTATTATTCCTGAAGTGGGCAATGCA

Consensus (551) CGATAAGTTTTGGGAAGAATGGAGCTATTATTCCTGAAGTGGGCAATGCA

601 650

GtCHI (598) GTAATAGAGAACAAGAAATTATCTGAAGCAGTGATAGAGTCTATTATTGG

GllCHI (520) GTAATAGAAAACAAGAAATTATCTGAAGCAGTTATAGAATCTATTATTGG

GlaCHI (520) GTAATAGAAAACAAGAAATTATCTGAAGCAGTTATAGAATCTATTATTGG

Consensus (601) GTAATAGAAAACAAGAAATTATCTGAAGCAGTTATAGAATCTATTATTGG

651 700

GtCHI (648) AGAGAAAGGTGTTTCACCAGCTGCAAAGAAGAGCTTGGCAACAAGAATTG

GllCHI (570) AGAGAAAGGTGTTTCACCAGCTGCAAAGAAGAGCTTGGCAACAAGAA

GlaCHI (570) AGAGAAAGGTGTTTCACCAGCTGCAAAGAAGAGCTTGGCAACAAGAA

Consensus (651) AGAGAAAGGTGTTTCACCAGCTGCAAAGAAGAGCTTGGCAACAAGAA

701 750

GtCHI (698) CAGAAATTTTGAACCATTTTGATGCT**TGA**TTAATATCAATTATGTTTATG

GllCHI (617)

GlaCHI (617)

Consensus (701)

**C**

301 350

GtF3H1 (296) ATTTGCCGCCGGAGGAAAAGCTCCGGTTTGATATGTCCGGTGGTAAGAAA

GllF3H1 (1) GTTTGATATGTCCGGTGGTAAGAAA

GlaF3H1 (1) GTTTGATATGTCCGGTGGTAAGAAA

GlaF3H2 (1) GTTTGATATGTCCGGTGGTAAGAAA

GllF3H2 (1) GTTTGATATGTCCGGTGGTAAGAAA

GtF3H2 (301) ATTTGCCGCCGGAGGAAAAGCTCCGGTTTGATATGTCCGGTGGTAAGAAA

Consensus (301) GTTTGATATGTCCGGTGGTAAGAAA

351 400

GtF3H1 (346) GGTGGATTCATTGTTTCCAGCCATTTGCAGGGAGAGGCGGTGCGAGATTG

GllF3H1 (26) GGTGGTTTCATTGTTTCCAGCCATTTGCAGGGAGAGGCAGTGCAAGATTG

GlaF3H1 (26) GGTGGTTTCATTGTTTCCAGCCATTTGCAGGGAGAGGCGGTGCAAGATTG

GlaF3H2 (26) GGTGGTTTCATTGTTTCCAGCCATTTGCAGGGAGAGGCGGTGCAAGATTG

GllF3H2 (26) GGTGGTTTCATTGTTTCCAGCCATTTGCAGGGAGAGGCAGTGCAAGATTG

GtF3H2 (351) GGTGGATTCATTGTTTCCAGCCATTTGCAGGGAGAGGCGGTGCGAGATTG

Consensus (351) GGTGGTTTCATTGTTTCCAGCCATTTGCAGGGAGAGGCGGTGCAAGATTG

401 450

GtF3H1 (396) GAGAGAAATTGTGACATACTTTTCCTACCCGATAAAATCAAGAGACTACT

GllF3H1 (76) GAGAGAAATAGTGACATACTTTTCCTACCCAATAAAATCAAGAGACTACA

GlaF3H1 (76) GAGAGAAATAATGATTTACTTTTCCTACCCAATAAAATCAAGAGACTACA

GlaF3H2 (76) GAGAGAAATAATGATTTACTTTTCCTACCCAATAAAATCAAGAGACTACA

GllF3H2 (76) GAGAGAAATAGTGACATACTTTTCCTACCCAATAAAATCAAGAGACTACA

GtF3H2 (401) GAGAGAAATTGTGACATACTTTTCCTACCCGATAAAATCAAGAGACTACT

Consensus (401) GAGAGAAATAGTGACATACTTTTCCTACCCAATAAAATCAAGAGACTACA

451 500

GtF3H1 (446) CGAGATGGCCAGATAAACCTGAAGGATGGAAATCCGTAACAGAAAAATAC

GllF3H1 (126) CAAGATGGCCAGATAAACCCGAAGGATGGAAATCCGTAACAGAAAAATAC

GlaF3H1 (126) CAAGATGGCCAGATAAACCCGAAGGTTGGAAATATGTAACAGAAAAATAC

GlaF3H2 (126) CAAGATGGCCAGATAAACCCGAAGGTTGGAAATATGTAACAGAAAAATAC

GllF3H2 (126) CAAGATGGCCAGATAAACCCGAAGGATGGAAATCCGTAACAGAAAAATAC

GtF3H2 (451) CGAGATGGCCAGATAAACCTGAAGGATGGAAATCCGTAACGGAAAAATAC

Consensus (451) CAAGATGGCCAGATAAACCCGAAGGATGGAAATCCGTAACAGAAAAATAC

501 550

GtF3H1 (496) AGTGAACAACTAATGAATCTTGCATGCAAATTGCTTGAAGTTCTGTCAGA

GllF3H1 (176) AGTGAACAACTAATGAATCTTGCATTCAAGCTACTTGAAGTTTTGTCAGA

GlaF3H1 (176) AGTGAGCAACTAATGAATCTTGCATGCAAGCTGCTTGAAGTTTTGTCAGA

GlaF3H2 (176) AGTGAGCAACTAATGAATCTTGCATGCAAGCTGCTTGAAGTTTTGTCAGA

GllF3H2 (176) AGTGAGCAACTAATGAATCTTGCATTCAAGCTGCTTGAAGTTTTGTCAGA

GtF3H2 (501) AGTGAACAGCTAATGAATCTTGCATGCAAATTGCTTGAAGTTCTGTCAGA

Consensus (501) AGTGAGCAACTAATGAATCTTGCATGCAAGCTGCTTGAAGTTTTGTCAGA

551 600

GtF3H1 (546) ATCAATGAGATTAGAAAAAGAGGCATTGACAAAGGCGTGTGTGGATATGG

GllF3H1 (226) ATCAATGGGATTAGAAAAAAAGGCAATGAAAAAGGCATGTGTGGATATGG

GlaF3H1 (226) ATCAATGGGACTAGAGAAAGAGGCATTGAAGAAGGCATGTGTGGATATGG

GlaF3H2 (226) ATCAATGGGACTAGAGAAAGAGGCATTGAAGAAGGCATGTGTGGATATGG

GllF3H2 (226) ATCAATGGGATTAGAAAAAAAGGCATTGAAAAAGGCATGTGTGGATATGG

GtF3H2 (551) ATCAATGAGATTAGAAAAGGAGGCATTGACGAAGGCGTGTGTGGATATGG

Consensus (551) ATCAATGGGATTAGAAAAAGAGGCATTGAAGAAGGCATGTGTGGATATGG

601 650

GtF3H1 (596) ATCAGAAAATAGTTGTGAACTTCTATCCGAAATGTCCACAACCTGACCTG

GllF3H1 (276) ATCAGAAAATAGTTGTGAATTTCTATCCAAAATGTCCACAACCTGATCTG

GlaF3H1 (276) ATCAGAAAATAGTTGTGAATTTCTATCCAAAATGTCCACAACCTGATCTG

GlaF3H2 (276) ATCAGAAAATAGTTGTGAATTTCTATCCAAAATGTCCACAACCTGATCTG

GllF3H2 (276) ATCAGAAAATAGTTGTGAATTTCTATCCGAAATGTCCACAACCTGATCTG

GtF3H2 (601) ATCAGAAAATAGTTGTGAACTTCTATCCGAAATGTCCACAACCTGACCTG

Consensus (601) ATCAGAAAATAGTTGTGAATTTCTATCCGAAATGTCCACAACCTGATCTG

651 700

GtF3H1 (646) ACACTTGGATTGAAACGGCATACCGATCCTGGCACCATAACGCTTTTGTT

GllF3H1 (326) ACACTGGGATTGAAACGACACACAGATCCTGGCACCATAACGTTGTTGTT

GlaF3H1 (326) ACACTGGGATTGAAACGACACACAGATCCTGGCACCATAACGTTGTTGTT

GlaF3H2 (326) ACACTGGGATTGAAACGACACACAGATCCTGGCACCATAACGTTGTTGTT

GllF3H2 (326) ACACTGGGATTGAAACGACACACAGATCCTGGCACCATAACGTTGTTGTT

GtF3H2 (651) ACACTTGGATTGAAACGGCATACCGATCCTGGCACCATAACGCTTTTGTT

Consensus (651) ACACTGGGATTGAAACGACACACAGATCCTGGCACCATAACGTTGTTGTT

701 750

GtF3H1 (696) ACAGGACCAGGTCGGGGGCCTTCAGGCCACCAGAGACGGCGGAAAAAGCT

GllF3H1 (376) ACAGGACCAGGTCGGCGGCCTTCAAGCCACCAGAGACGGGGGCAAGAGTT

GlaF3H1 (376) ACAGGACCAGGTCGGCGGCCTTCAAGCCACCAGAGACGGGGGCAATAGTT

GlaF3H2 (376) ACAGGACCAGGTCGGCGGCCTTCAAGCCACCAGAGACGGGGGCAAGAGTT

GllF3H2 (376) ACAGGACCAGGTCGGCGGCCTTCAAGCCACCAGAGACGGGGGCAAGAGTT

GtF3H2 (701) ACAGGACCAGGTCGGGGGCCTTCAGGCCACCAGAGACGGCGGAAAAAGCT

Consensus (701) ACAGGACCAGGTCGGCGGCCTTCAAGCCACCAGAGACGGGGGCAAGAGTT

751 800

GtF3H1 (746) GGATCACTGTTCAGCCTGTTGATGGTGCTTTTGTTGTTAACCTCGGTGAC

GllF3H1 (426) GGATCACTGTGATGCCTGTTGATGGTGCTTTTGTTGTCAACCTCGGTGAC

GlaF3H1 (426) GGATCACTGTGATGCCTGTTGATGGTGCTTTTGTTGTCAACCTCGGTGAC

GlaF3H2 (426) GGATCACTGTGATGCCTGTTGATGGTGCTTTTGTTGTCAACCTCGGTGAC

GllF3H2 (426) GGATCACTGTGATGCCTGTTGATGGTGCTTTTGTTGTCAACCTCGGTGAC

GtF3H2 (751) GGATCACTGTTCAGCCTGTTGATGGTGCTTTTGTTGTTAACCTCGGTGAC

Consensus (751) GGATCACTGTGATGCCTGTTGATGGTGCTTTTGTTGTCAACCTCGGTGAC

801 850

GtF3H1 (796) CATGGACATTACTTAAGCAACGGGAGGTTCAAGAACGCTGACCACCAAGC

GllF3H1 (476) CATGGACATTACTTGAGCAATGGGAGGTTCAAGAATGCAGACCACCAAGC

GlaF3H1 (476) CATGGACATTACTTGAGCAATGGGAGGTTCAAGAATGCAGACCACCAAGC

GlaF3H2 (476) CATGGACATTACTTGAGCAATGGGAGGTTCAAGAATGCAGACCACCAAGC

GllF3H2 (476) CATGGACATTACTTGAGCAATGGGAGGTTCAAGAATGCAGACCACCAAGC

GtF3H2 (801) CATGGACATTACTTAAGCAACGGGAGGTTCAAGAACGCTGACCACCAAGC

Consensus (801) CATGGACATTACTTGAGCAATGGGAGGTTCAAGAATGCAGACCACCAAGC

851 900

GtF3H1 (846) TGTAGTGAACTCAAACTACAGTAGGCTATCCATTGCCACATTTCAGAATC

GllF3H1 (526) AGTGGTGAACTCAAACTGCAGTAGGCTATCCATTGCCACATTTCAGAATC

GlaF3H1 (526) AGTGGTGAACTCAAACTGCAGTAGGCTATCCATTGCCACATTTCAGAATC

GlaF3H2 (526) AGTGGTGAACTCAAACTGCAGTAGGCTATCCATTGCCACATTTCAGAATC

GllF3H2 (526) AGTGGTGAACTCAAACTGCAGTAGGCTATCCATTGCCACATTTCAGAATC

GtF3H2 (851) TGTAGTGAACTCAAACTACAGTAGGCTATCCATTGCCACATTTCAGAATC

Consensus (851) AGTGGTGAACTCAAACTGCAGTAGGCTATCCATTGCCACATTTCAGAATC

901 950

GtF3H1 (896) CGGCACCGGAGGCCACCGTATACCCGCTGGCGATAAGGGACGGCGAGAAG

GllF3H1 (576) CGGCGCCGGAGGCGACTGTATACCCGTTGGCGGTAAGGGACGGTGAGAAG

GlaF3H1 (576) CGGCGCCGGAGGCGACTGTATACCCGTTGGCGGTAAGGGACGGTGAGAAG

GlaF3H2 (576) CGGCGCCGGAGGCGACTGTATACCCGTTGGCGGTAAGGGACGGTGAGAAG

GllF3H2 (576) CGGCGCCGGAGGCGACTGTATACCCGTTGGCGGTAAGGGACGGTGAGAAG

GtF3H2 (901) CGGCACCGGAGGCCACCGTATACCCGCTGGCGATAAGGGACGGCGAGAAG

Consensus (901) CGGCGCCGGAGGCGACTGTATACCCGTTGGCGGTAAGGGACGGTGAGAAG

951 1000

GtF3H1 (946) CCGGTTCTCGACGAGCCGATTACGTTCGCCGAGATGTACCGGAGGAAGAT

GllF3H1 (626) CCGGTTCTCGATGAGCCGATAACGTTCGCCGAGATGTACCGGAGGAAGAT

GlaF3H1 (626) CCGGTTCTCGATGAGCCGATAACGTTCGCCGAGATGTACCGGAGGAAGAT

GlaF3H2 (626) CCGGTTCTCGATGAGCCGATTACGTTCGCCGAGATGTACCGGAGGAAGAT

GllF3H2 (626) CCGGTTCTCGATGAGCCGATTACGTTCGCCGAGATGTACCGGAGGAAGAT

GtF3H2 (951) CCGGTTCTCGACGAGCCGATTACGTTCGCTGAGATGTACCGGAGGAAGAT

Consensus (951) CCGGTTCTCGATGAGCCGATTACGTTCGCCGAGATGTACCGGAGGAAGAT

1001 1050

GtF3H1 (996) GAGCAAAGATCTGGAACTTGCAAGGTTGAAGAAGCAAGCAAAAGAGGAGT

GllF3H1 (676) GAGCAAAGATCTTGAACTTGCAAGGTTGAAG---CAAGCAAAAGAGGAGT

GlaF3H1 (676) GAGCAAAGATCTTGAACTTGCAAGGTTGAAG---CAAGCAAAAGAGGAGT

GlaF3H2 (676) GAGCAAAGATCTTGAACTTGCAAGGTTGAAGAAGCAAGCAAAAGAGGAGT

GllF3H2 (676) GAGCAAAGATCTTGAACTTGCAAGGTTGAAGAAGCAAGCAAAAGAGGAGT

GtF3H2 (1001) GAGCAAAGATCTGGAACTTGCAAGGTTGAAGAAGCAAGCAAAAGAGGAGT

Consensus (1001) GAGCAAAGATCTTGAACTTGCAAGGTTGAAGAAGCAAGCAAAAGAGGAGT

1051 1100

GtF3H1 (1046) TGAAAAATGTTGAAAAGGCTAAAATTGAAGGGAAGGCTCTTGAGGAGATT

GllF3H1 (723) TGAAAAATGTTGAAACTGCT**TAA**ATTGTGGGTAAGCCTATTGAGGAGATT

GlaF3H1 (723) TGAAAAATGTTGAAACTGCT**TAA**ATTGTGGGTAAGCCTATTGAGGAGATT

GlaF3H2 (726) TGAAAAATGTTGAAACTGCT**TAA**ATTGTGGGTAAGCCTATTGAGGAGATT

GllF3H2 (726) TGAAAAATGTTGAAACTGCT**TAA**ATTGTGGGTAAGCCTATTGAGGAGATT

GtF3H2 (1051) TGAAAAATGTTGAAAAGGCTAAAATTGAAGGGAAGGCTCTTGAGGAGATT

Consensus (1051) TGAAAAATGTTGAAACTGCTTAAATTGTGGGTAAGCCTATTGAGGAGATT

1101 1150

GtF3H1 (1096) CTTGCT**TAA**ACTTTTGGTACCCGAGCAATGTTACGGTCATAACAACAAAC

GllF3H1 (773) CTTGCTTAA--TTTTGGTACC-GAGTTATATTACGGTCATAACAATGAAC

GlaF3H1 (773) CTTGCTTAA--TTTTGGTACC-GAGTTATATTACGGTCATAACAATGAAC

GlaF3H2 (776) CTTGCTTAAACTTTTGGTACC-GAGTTATATTACGGTCATAACAATGAAC

GllF3H2 (776) CTTGCTTAAACTTTTGGTACC-GAGTTATATTACGGTCATAACAATGAAC

GtF3H2 (1101) CTTGCT**TAA**ACTTTTGGTACCCGAGCAACGTTACGGTCATAACAACAAAC

Consensus (1101) CTTGCTTAAACTTTTGGTACC GAGTTATATTACGGTCATAACAATGAAC

1151 1200

GtF3H1 (1146) ACGATGTGATGACGCGCGTGGTTGTGGGCCCACGCATATTAGGTTTTTT-

GllF3H1 (820) ATAAGTTGATGAT----GTGACAGTGAGTCCATGTAAATTAGTTTTTTTT

GlaF3H1 (820) ATAAGTTGATGAT----GTGGCCGTGAGTCCATGTAAATTAGTTTTTTTT

GlaF3H2 (825) ATAAGTTGATGAT----GTGGCAGTGAGTCCATATAAATTAGTTTTTTTT

GllF3H2 (825) ATAAGTTGATGAT----GTGGCAGTGAGTCCATATAAATTAGTTTTTTTT

GtF3H2 (1151) ACGATGTGATGACGCGCGTGGTTGTGGGCCCACGCATATTAGGTTTTTT-

Consensus (1151) ATAAGTTGATGAT GTGGCAGTGAGTCCATGTAAATTAGTTTTTTTT

1201 1250

GtF3H1 (1195) AATACACGGAACTTATTGACACGTCATGTTGTGCATGTCCGCTGTTATGG

GllF3H1 (866) AACACATGAAACCCACTGACATGTGATGTTGT----GTCCACTGTTATGA

GlaF3H1 (866) AACACATGGAACCCACTGACACGTGATGTTGT----GTCCACTGTTATGA

GlaF3H2 (871) AACACATGGAACTCACTGACACGTGATGTTGT----GTCCACTGTTATGA

GllF3H2 (871) AACACATGGAACTCACTGACACGTGATGTTGT----GTCCACTGTTATGA

GtF3H2 (1200) AATACACGGAACTGATTGACACGTCATGTTGTGCATGTCCGTTGTTATGG

Consensus (1201) AACACATGGAACTCACTGACACGTGATGTTGT GTCCACTGTTATGA

1251 1300

GtF3H1 (1245) AAGATTTGTCGTAGCTGTTGTGTTACTCGATCGTTTCTATATTTATATTT

GllF3H1 (912) CAGACTTGTTGCAGCA--CGTATCACT------TATTGATATTTCTAGTT

GlaF3H1 (912) CAGACTTGTTGCAGCA--CGTATCACT------TATTGATATTTCTAGTT

GlaF3H2 (917) CAGACTTGTTGCAGCA--CGTATCACT------TATTGATATTTCTAGTT

GllF3H2 (917) CAGACTTGTTGCAGCA--CGTATCACT------TATTGATATTTCTAGTT

GtF3H2 (1250) AAGATTTGTCGTAGCTGTTGTGTTACTCGATCGTTTCTATATTTATATTT

Consensus (1251) CAGACTTGTTGCAGCA CGTATCACT TATTGATATTTCTAGTT

1301 1350

GtF3H1 (1295) ATGTTATAATTTGCATCGCATTATATATTTTATAATATTTAATGGTGTTA

GllF3H1 (954) ATGTTATAATTTGCATCGCATTATATATTTTATAATATTTAATG

GlaF3H1 (954) ATGTTATAATTTGCATCGCATTATATATTTTATAATATTTAATG

GlaF3H2 (959) ATGTTATAATTTGCATCGCATTATATATTTTATAATATTTAATG

GllF3H2 (959) ATGTTATAATTTGCATCGCATTATATATTTTATAATATTTAATG

GtF3H2 (1300) ATGTTATAATTTGCATCGCATTATATATTTTATAATATTTAATGGTGTTA

Consensus (1301) ATGTTATAATTTGCATCGCATTATATATTTTATAATATTTAATG

**D**

1 50

GtDFR (1) CTAATCTTCAAGCCTGGAA**ATG**GAAGGAGGGATTTTATCAAATGCCACAA

GllDFR1 (1) **ATG**GAAGGAGGGATTTTATCAAATGCCACAA

GlaDFR1 (1) **ATG**GAAGGAGGGATTTTATCAAATGCCACAA

GlaDFR2 (1) **ATG**GAAGGAGGGATTTTATCAAATGCCACAA

GllDFR2 (1) **ATG**GAAGGAGGGATTTTATCAAATGCCACAA

Consensus (1) ATGGAAGGAGGGATTTTATCAAATGCCACAA

51 100

GtDFR (51) CTGTATGTGTTACCGGAGCTTCTGGATATATCGGATCATGGCTAGCCATG

GllDFR1 (32) CTGTATGTGTTACTGGAGCTTCTGGATACGTTGGATCGTGGCTAGTCATG

GlaDFR1 (32) CTGTATGTGTTACTGGAGCTTCTGGATACGTTGGATCGTGGCTAGTCATG

GlaDFR2 (32) CTGTATGTGTTACTGGAGCTGCTGGATATATTGGATCGTGGCTAGTCATG

GllDFR2 (32) CTGTATGTGTTACTGGAGCTGCTGGATATATTGGATCGTGGCTAGTCATG

Consensus (51) CTGTATGTGTTACTGGAGCTTCTGGATATATTGGATCGTGGCTAGTCATG

101 150

GtDFR (101) AGACTTCTTGAACGCGGTTATACTGTTCGTGCCACTGTTCGGGATCCCGG

GllDFR1 (82) AGACTTCTGGAACGTGGTTATACTGTACGTGCCACTGTTCGTGATCCTGC

GlaDFR1 (82) AGACTTCTGGAACGTGGTTATACTGTACGTGCCACTGTTCGTGATCCTGC

GlaDFR2 (82) AGACTTCTGGAACGTGGTTATACTGTTCGTGCCACTGTTCGTGATCCTGG

GllDFR2 (82) AGACTTCTGGAACGTGGTTATACTGTTCGTGCCACTGTTCGTGATCCTGG

Consensus (101) AGACTTCTGGAACGTGGTTATACTGTTCGTGCCACTGTTCGTGATCCTGG

151 200

GtDFR (151) GAATCTGAAGAAGGTTCAACATCTTCTAGAGCTACCAAAAGCCAGCACGA

GllDFR1 (132) GAATCTGAAGAAAGTTCAACATCTTCTAGAGTTACCAAAAGCCAGCACAA

GlaDFR1 (132) GAATCTGAAGAAAGTTCAACATCTTCTAGAGTTACCAAAAGCCAGCACAA

GlaDFR2 (132) GAATCAGAAGAAGGTTAAACATCTACTAGAGCTACCAAAGGCCAGCACAA

GllDFR2 (132) GAATCAGAAGAAGGTTAAACATCTACTAGAGCTACCAAAGGCCAGCACAA

Consensus (151) GAATCTGAAGAAGGTTCAACATCTTCTAGAGCTACCAAAAGCCAGCACAA

201 250

GtDFR (201) ATTTGACGTTGTTGAAGGCGGACTTGACAGAAGAAGGAAGCTTTGATGAA

GllDFR1 (182) ATTTGACGTTGTTGAAGGCGGACTTGACAGAAGAAGGAAGCTTTGATGAA

GlaDFR1 (182) ATTTGACGTTGTTGAAGGCGGACTTGACAGAAGAAGGAAGCTTTGATGAA

GlaDFR2 (182) ATTTGACGTTATTGAAGGCGGACTTGACAGAAGAAGGAAGCTTTGATGAA

GllDFR2 (182) ATTTGACGTTATTGAAGGCGGACTTGACAGAAGAAGGAAGCTTTGATGAA

Consensus (201) ATTTGACGTTGTTGAAGGCGGACTTGACAGAAGAAGGAAGCTTTGATGAA

251 300

GtDFR (251) GCCATTCATGGCTGTCATGGTGTTTTTCACGTGGCTACTCCCATGGACTT

GllDFR1 (232) GCCATTCATGGCTGTCATGGAGTTTTTCATGTGGCTACTCCAATGGACTT

GlaDFR1 (232) GCCATTCATGGCTGTCATGGAGTTTTTCATGTGGCTACTCCAATGGACTT

GlaDFR2 (232) GCCATTCATGGCTGTCATGGAGTTTTTCATGTGGCTACTCCCATGGACTT

GllDFR2 (232) GCCATTCATGGCTGTCATGGAGTTTTTCATGTGGCTACTCCCATGGACTT

Consensus (251) GCCATTCATGGCTGTCATGGAGTTTTTCATGTGGCTACTCCCATGGACTT

301 350

GtDFR (301) CGAATCCAAAGACCCCAAGAATGAAGTGATCAAACCAACAATTGATGGGT

GllDFR1 (282) CGAATCCAAAGACCCCGAGAATGAAGTGATCAAACCAACCATTGATGGGT

GlaDFR1 (282) CGAATCCAAGGACCCCGAGAATGAAGTGATCAAACCAACCATTGATGGGT

GlaDFR2 (282) CGAATCCAAGGACCCTGAGAATGAAGTGATCAAACCAACGATTGATGGGT

GllDFR2 (282) CGAATCCAAGGACCCTGAGAATGAAGTGATCAAACCAACGATTGATGGGT

Consensus (301) CGAATCCAAGGACCCCGAGAATGAAGTGATCAAACCAAC ATTGATGGGT

351 400

GtDFR (351) TTTTGAGCATTATAAGGTCGTGTGTAAAGGCAAAGACAGTGAAGAAGCTG

GllDFR1 (332) TTTTAAGCATTATAAGGTCATGTGTAAAGGCAAAGACAGTGAAGAAGCTA

GlaDFR1 (332) TTTTAAGCATTATAAGGTCATGTGTAAAGGCAAAGACAGTGAAGAAGCTA

GlaDFR2 (332) TTCTGAGCATTATACAGTCGTGTGTAAAGGCAAAGACAGTGAAGAAGCTA

GllDFR2 (332) TTCTGAGCATTATACAGTCGTGTGTAAAGGCAAAGACAGTGAAGAAGCTA

Consensus (351) TTTTGAGCATTATAAGGTCGTGTGTAAAGGCAAAGACAGTGAAGAAGCTA

401 450

GtDFR (401) GTTTTCACATCATCTGCTGGAACTGTTGATGTTCAAGAACAACAGAAACC

GllDFR1 (382) GTCTTCACATCATCTGCTGGTACCGTTGATGTTCAACAACAACAGAAACC

GlaDFR1 (382) GTCTTCACATCATCTGCTGGTACCGTTGATGTTCAACAACAACAGAAACC

GlaDFR2 (382) GTCTTCACATCATCTGCTGGAACCGTTGATGCTCAAGAACAACAGAAACC

GllDFR2 (382) GTCTTCACATCATCTGCTGGAACCGTTGATGCTCAAGAACAACAGAAACC

Consensus (401) GTCTTCACATCATCTGCTGGAACCGTTGATGTTCAAGAACAACAGAAACC

451 500

GtDFR (451) AGTATACGATGAGAACGATTGGAGTGACTTGGATTTCATCAACTCCACTA

GllDFR1 (432) CGTCTACGACGAGAACGACTGGAGTGACTTGGATTTCATTAACTCCAATA

GlaDFR1 (432) CGTCTACGACGAGAACGACTGGAGTGACTTGGATTTCATTAACTCCAATA

GlaDFR2 (432) TGTCTATGACGAGGACGACTGGAGTGACCTGGATTTCATCAAGTCCAATA

GllDFR2 (432) TGTCTATGACGAGGACGACTGGAGTGACCTGGATTTCATCAAGTCCAATA

Consensus (451) GTCTACGACGAGAACGACTGGAGTGACTTGGATTTCATCAACTCCAATA

501 550

GtDFR (501) AAATGACCGGATGGATGTATTTTGTCTCCAAAATACTGGCAGAAAAAGCA

GllDFR1 (482) AAATGACCGGATGGATGTATTTTGTCTCCAAAATACTGGCAGAGAAGGCA

GlaDFR1 (482) AAATGACCGGATGGATGTATTTTGTCTCCAAAATACTGGCAGAGAAGGCA

GlaDFR2 (482) AAATGACTGGATGGATGTATTTTGTCTCCAAAATACTGGCAGAGAAAGCA

GllDFR2 (482) AAATGACTGGATGGATGTATTTTGTCTCCAAAATACTGGCAGAGAAAGCA

Consensus (501) AAATGACCGGATGGATGTATTTTGTCTCCAAAATACTGGCAGAGAAAGCA

551 600

GtDFR (551) GCATGGGAAGTAACTAAAGCAAACGACATCGGTTTTATTAGCATCATTCC

GllDFR1 (532) GCATGGGAAGCAGCTAAAGCAAACAACATTGGTTTCATCAGCATCATTCC

GlaDFR1 (532) GCATGGGAAGCAGCTAAAGCAAACAACATTGGTTTCATCAGCATCATTCC

GlaDFR2 (532) GCATGGGAAGCAGCTAAAGCAAACAACATCAGTTTCATTAGCATCATTCC

GllDFR2 (532) GCATGGGAAGCAGCTAAAGCAAACAACATCAGTTTCATTAGCATCATTCC

Consensus (551) GCATGGGAAGCAGCTAAAGCAAACAACATCGGTTTCATTAGCATCATTCC

601 650

GtDFR (601) AACGTTAGTCGTTGGTCCATTCATCACGACAACATTCCCACCCAGCCTAA

GllDFR1 (582) AACGTTAGTCGTTGGTCCATTCATCACGCCTACATTCCCACCCAGCCTAA

GlaDFR1 (582) AACGTTAGTCGTTGGTCCATTCATCACGCCTACATTCCCACCCAGCCTAA

GlaDFR2 (582) AACGTTAGTCGTTGGTCCATTCATCACGCCCACATTCCCACCCAGCCTAA

GllDFR2 (582) AACGTTAGTCGTTGGTCCATTCATCACGCCCACATTCCCACCCAGCCTAA

Consensus (601) AACGTTAGTCGTTGGTCCATTCATCACGCC ACATTCCCACCCAGCCTAA

651 700

GtDFR (651) TCACCGCGCTCTCATTGATTACCGGGAATGAAGCACACTACGGTATCATT

GllDFR1 (632) TTACTGCCCTCTCATTGATCACCGGGAATGAAGCACACTATGGTATCATT

GlaDFR1 (632) TTACTGCCCTCTCATTGATCACCGGGAATGAAGCACACTATGGTATCATT

GlaDFR2 (632) TTACCGCCCTCTCATTGATCACCGGGAATGAAGCACACTATGGTATCATT

GllDFR2 (632) TTACCGCCCTCTCATTGATCACCGGGAATGAAGCACACTATGGTATCATT

Consensus (651) TTACCGCCCTCTCATTGATCACCGGGAATGAAGCACACTATGGTATCATT

701 750

GtDFR (701) AAACAAGGCCAATTTGTGCATCTAGATGATCTCTGTGAAGCTCATATATT

GllDFR1 (682) AAGCAAGGCCAATTTGTGCATCTAGATGACCTCTGTGAAGCTCATATATT

GlaDFR1 (682) AAGCAAGGCCAATTTGTGCATCTAGATGACCTCTGTGAAGCTCATATATT

GlaDFR2 (682) AAGCAAGGCCAATTTGTGCATCTAGATGACCTCTGTGAAGCTCATATATT

GllDFR2 (682) AAGCAAGGCCAATTTGTGCATCTAGATGACCTCTGTGAAGCTCATATATT

Consensus (701) AAGCAAGGCCAATTTGTGCATCTAGATGACCTCTGTGAAGCTCATATATT

751 800

GtDFR (751) TTTATATGAACATCCCGAAGCCGAAGGAAGATACATTTGCTCATCTCATG

GllDFR1 (732) TTTATATGAACACCCAGAAGCAGAAGGAAGATACATTTGCTCTTCTCATG

GlaDFR1 (732) TTTATATGAACACCCAGAAGCAGAAGGAAGATACATTTGCTCTTCTCATG

GlaDFR2 (732) TTTATATGAACACCCAGAAGCAGAAGGAAGATACATTTGCTCTTCTCATG

GllDFR2 (732) TTTATATGAACACCCAGAAGCAGAAGGAAGATACATTTGCTCTTCTCATG

Consensus (751) TTTATATGAACACCCAGAAGCAGAAGGAAGATACATTTGCTCTTCTCATG

801 850

GtDFR (801) ACACAACCATCCATGATTTGGCAAAAATGATCAGACAAAATTGGCCAGAA

GllDFR1 (782) ACACAACCATCAATGATTTGGCAAAAATGATCAGAGATAAATGGCCACAA

GlaDFR1 (782) ACACAACCATCAATGATTTGGCAAAAATGATCAGAGATAAATGGCCACAA

GlaDFR2 (782) ACACAACCATCAATGATTTGGCAAAAATGATCAGAGATAAATGGCCACAA

GllDFR2 (782) ACACAACCATCAATGATTTGGCAAAAATGATCAGAGATAAATGGCCACAA

Consensus (801) ACACAACCATCAATGATTTGGCAAAAATGATCAGAGATAAATGGCCACAA

851 900

GtDFR (851) TACTACATTCCCACTAAGTTAAAGGGAATTGATGAAGACATACCTGTGGT

GllDFR1 (832) TACTACATCCCCACTAAGTTAAAGGGAATTGATGAAGACATACCTGTGGT

GlaDFR1 (832) TACTACATCCCCACTAAGTTAAAGGGAATTGATGAAGACATACCTGTGGT

GlaDFR2 (832) TACTACATCCCCACTAAGTTAAAGGGAATTGATGAAGACATACCTGTGGT

GllDFR2 (832) TACTACATCCCCACTAAGTTAAAGGGAATTGATGAAGACATACCTGTGGT

Consensus (851) TACTACATCCCCACTAAGTTAAAGGGAATTGATGAAGACATACCTGTGGT

901 950

GtDFR (901) ATCCTTTTCATCAAATAAATTGATAGATTTGGGTTTTCAATACAAATACA

GllDFR1 (882) ATCCTTTTCATCAAAGAAACTGACTGAGATGGGTTTTCAATACAAATATA

GlaDFR1 (882) ATCCTTTTCATCAAAGAAACTGACTGAGATGGGTTTTCAATACAAATATA

GlaDFR2 (882) ATCCTTTTCATCAAAGAAACTGACTGAGATGGGTTTTCAATACAAATATA

GllDFR2 (882) ATCCTTTTCATCAAAGAAACTGTCTGAGATGGGTTTTCAATCCAAATATA

Consensus (901) ATCCTTTTCATCAAAGAAACTGACTGAGATGGGTTTTCAATACAAATATA

951 1000

GtDFR (951) CCCTGGAAGACATGTTCAGAGGAGCCATTGATACATGCAAAGAGAAAAGG

GllDFR1 (932) GCTTGGAAGATATGTTCAGAGGAGCCATTGATACATGCAAAGAGAAAGGG

GlaDFR1 (932) GCTTGGAAGATATGTTCAGAGGAGCCATTGATACATGCAAAGAGAAAGGG

GlaDFR2 (932) GCTTGGAAGATATGTTCAGAGGAGCCATTGATACATGCAAAGAGAAAGGG

GllDFR2 (932) GCTTGGAAGACATGTTCAGAGGAGCCATTGATACATGCAAAGAGAAAGGG

Consensus (951) GCTTGGAAGATATGTTCAGAGGAGCCATTGATACATGCAAAGAGAAAGGG

1001 1050

GtDFR (1001) ATGCTTCCACTTTCTATTGGGCACCAAAAAGAATCTACTGACCCAGAAGT

GllDFR1 (982) ATGCTTCCACATTCTACTGGGCACAAAAAAGAATCCACTGAGCCAGAAAT

GlaDFR1 (982) ATGCTTCCACATTCTACTGGGCACAAAAAAGAATCCACTGACCCAGAAAT

GlaDFR2 (982) ATGCTTCCACATTCTACTGGGCACCAAAAAGAATCCACTGACACAGAAGT

GllDFR2 (982) ATGCTTCCACATTCTACTGGGCACCAAAAAGAATCCACTGACACAGAAGT

Consensus (1001) ATGCTTCCACATTCTACTGGGCACCAAAAAGAATCCACTGACCCAGAAGT

1051 1100

GtDFR (1051) TGACGAGGTTGTAAAAGAGATGGAACTGATTCAAGATTCACTAGAC**TAG**A

GllDFR1 (1032) CGAAGACCCT--AA---GGATGGAACAA----AAGAAGCAA-ATTCTGAA

GlaDFR1 (1032) CGAAGACCCT--AA---GGATGGAACAA----AAGAAGCAA-ATTCTGAA

GlaDFR2 (1032) CAAAGACCCT--AA---GGATGGAACAA----AAGAAGCAA-ATTCTGAA

GllDFR2 (1032) CAAAGACCCT--AA---GGATGGAACAA----AAGAAGCAA-ATTCTGAA

Consensus (1051) CGAAGACCCT AA GGATGGAACAA AAGAAGCAA ATTCTGAA

1101 1150

GtDFR (1101) AGTTTGTAACAAAT-GAACAAGTACTGGCGTATTGAGAAGAAAGCTCAGC

GllDFR1 (1072) AATCATCAAGAGATTGTAAAAGAGATGG--AACTGATTCAAGAT-TCACT

GlaDFR1 (1072) AATCATCAAGAGATTGTAAAAGAGATGG--AACTGATTCAAGAT-TCACT

GlaDFR2 (1072) AATCATCAAGAGATTGTAAAAGAGATGG--AACTGATTCAAGAT-TCACT

GllDFR2 (1072) AATCATCAAGAGGTTGTAAAAGAGATGG--AACTGATTCAAGAT-TCACT

Consensus (1101) AATCATCAAGAGATTGTAAAAGAGATGG AACTGATTCAAGAT TCACT

1151 1200

GtDFR (1150) AAAGTATTTTCATTTATATTTCTATAATAATTATGAATAGCCCAAAAAAG

GllDFR1 (1119) AGAC**TAG**A

GlaDFR1 (1119) AGAC**TAG**A

GlaDFR2 (1119) AGAC**TAG**A

GllDFR2 (1119) AGAC**TAG**A

Consensus (1151) AGACTAGA

**E**

1 50

GtANS (1) ATATA**ATG**GGATCTCTTTTGCCTAGTAGAGTTGAAAGCTTAGCCATCAGT

GllANS1 (1) **ATG**GGATCTCTTTTGCCTAGTAGAGTTGAAAGCTTGGCCATGAGT

GlaANS1 (1) **ATG**GGATCTCTTTTGCCTAGTAGAGTTGAAAGCTTGGCCATGAGT

GlaANS2 (1) **ATG**GGATCTCTTTTGCCTAGTAGAGTTGAAAGCTTGGCCATGAGT

GllANS2 (1) **ATG**GGATCTCTTTTGCCTAGTAGAGTTGAAAGCTTGGCCATGAGT

Consensus (1) ATGGGATCTCTTTTGCCTAGTAGAGTTGAAAGCTTGGCCATGAGT

51 100

GtANS (51) GGTATCAAAACAATTCCAAAAGAATACATTAGGCCTAAAGAAGAACTTGC

GllANS1 (46) GGTATCAAAACAATTCCAAAAGAATACGTGAGGCCTAAAGAAGAAGTGGC

GlaANS1 (46) GGTATCAAAACAATTCCAAAAGAATACGTGAGGCCTAAAGAAGAAGTGGC

GlaANS2 (46) TGTATCAAAACAATTCCAAGAGAATACGTGAGGCCTAAAGAAGAAGTGGC

GllANS2 (46) TGTATCAAAACAATTCCAAGAGAATACGTGAGGCCTAAAGAAGAAGTGGC

Consensus (51) GGTATCAAAACAATTCCAAAAGAATACGTGAGGCCTAAAGAAGAAGTGGC

101 150

GtANS (101) AAGCATTGGAAACATTTTCGAAGAAGCGAAAAACAACAACAAAACTAGCC

GllANS1 (96) AAGCATTGGAAATGTTTTCGAAGAAGATGAAACC-----CAAACCCAG--

GlaANS1 (96) AAGCATTGGAAATGTTTTCGAAGAAGATGAAACC-----CAAACCCAG--

GlaANS2 (96) AAGCATTGGAAATGTTTTCGAAGAAGATGAAACC-----CAAACCCAG--

GllANS2 (96) AAGCATTGGAAATGTTTTCGAAGAAGATGAAACC-----CAAACCCAG--

Consensus (101) AAGCATTGGAAATGTTTTCGAAGAAGATGAAACC CAAACCCAG

151 200

GtANS (151) AGATAGTACCAACCATCGATTTAAAAGACATGGATTCATTAGACAACAAC

GllANS1 (139) -----GTACCAACCATCGATTTAAAAGACTTGGATTCATTGGACGACAA-

GlaANS1 (139) -----GTACCAACCATCGATTTAAAAGACTTGGATTCATTGGACGACAA-

GlaANS2 (139) -----GTACCAACCATCGATTTAAAAGACTTGGATTCATTAGACAACAA-

GllANS2 (139) -----GTACCAACCATCGATTTAAAAGACTTGGATTCATTAGACAACAA-

Consensus (151) GTACCAACCATCGATTTAAAAGACTTGGATTCATTAGACAACAA

201 250

GtANS (201) AAAGATGTTCAGACACAATGTCACGACGAATTAAAGAACGCCGCAATGGA

GllANS1 (183) --AGATGTTCAGACAAGATGTCACGACGAATTAAAGAAAGCTGCTATGGA

GlaANS1 (183) --AGATGTTCAGACAAGATGTCACGACGAATTAAAGAAAGCTGCTATGGA

GlaANS2 (183) --AGATGTTCAGACAAGATGTCACGACGAATTAAAGAAAGCTGCTATGGA

GllANS2 (183) --AGATGTTCAGACAAGATGTCACGACGAATTAAAGAAAGCTGCTATGGA

Consensus (201) AGATGTTCAGACAAGATGTCACGACGAATTAAAGAAAGCTGCTATGGA

251 300

GtANS (251) ATGGGGAGTGATGAACCTTGTCAACCACGGTATATCGCAAGAACTCATTA

GllANS1 (231) ATGGGGAGTGATGCACCTTGTCAACCATGGTATATCATATGATATCATTA

GlaANS1 (231) ATGGGGAGTGATGCACCTTGTCAACCATGGTATATCATATGATATCATTA

GlaANS2 (231) ATGGGGAGTGATGCACCTTGTCAACCATGGTATATCCTATGATATCATTA

GllANS2 (231) ATGGGGAGTGATGCACCTTGTCAACCATGGTATATCCTATGATATCATTA

Consensus (251) ATGGGGAGTGATGCACCTTGTCAACCATGGTATATC TATGATATCATTA

301 350

GtANS (301) ATCGCGTAAAATCCGCAGGACAAGCCTTCTTTGATCTTCCTATTGAAGAA

GllANS1 (281) ATCGCGTCAAATCTGCAGGACAAGCCTTCTTTGATCTTCCTATTGAGGAA

GlaANS1 (281) ATCGCGTCAAATCTGCAGGACAAGCCTTCTTTGATCTTCCTATTGAGGAA

GlaANS2 (281) ATCGCGTCAAATCTGCAGGACAAGCCTTCTTTGATCTTCCTATTGAGGAA

GllANS2 (281) ATCGCGTCAAATCTGCAGGACAAGCCTTCTTTGATCTTCCTATTGAGGAA

Consensus (301) ATCGCGTCAAATCTGCAGGACAAGCCTTCTTTGATCTTCCTATTGAGGAA

351 400

GtANS (351) AAAGAGAAGTATGCAAATGATCAGGCCTCTGGGAATGTGCAGGGCTATGG

GllANS1 (331) AAAGAGAAGTATGCCAATGATCAAGCCTCTGGGAATGTCCAGGGTTATGG

GlaANS1 (331) AAAGAGAAGTATGCCAATGATCAAGCCTCTGGGAATGTCCAGGGTTATGG

GlaANS2 (331) AAAGAGAAGTATGCCAATGATCAAGCCTCTGGGAATGTCCAGGGTTATGG

GllANS2 (331) AAAGAGAAGTATGCCAATGATCAAGCCTCTGGGAATGTCCAGGGTTATGG

Consensus (351) AAAGAGAAGTATGCCAATGATCAAGCCTCTGGGAATGTCCAGGGTTATGG

401 450

GtANS (401) TAGTAGGCTTGCTAATAATGCAAGTGGTCAGCTGGAATGGGAGGATTATT

GllANS1 (381) TAGCAGGCTTGCTAATAATGCAAGTGGTCAGCTTGAATGGGAGGATTACT

GlaANS1 (381) TAGCAGGCTTGCTAATAATGCAAGTGGTCAGCTTGAATGGGAGGATTACT

GlaANS2 (381) TAGCAGGCTTGCTAATAATGCAAGTGGTCAGCTTGAATGGGAGGATTACT

GllANS2 (381) TAGCAGGCTTGCTAATAATGCAAGTGGTCAGCTTGAATGGGAGGATTACT

Consensus (401) TAGCAGGCTTGCTAATAATGCAAGTGGTCAGCTTGAATGGGAGGATTACT

451 500

GtANS (451) TTTTTCATTGTATTTACCCTGAAAGGAAAAGGGACATGTCTATCTGGCCA

GllANS1 (431) TTTTTCATTGTATTTACCCTCAAGGGAAACGCGACATGTCTATCTGGCCA

GlaANS1 (431) TTTTTCATTGTATTTACCCTCAAGGGAAACGCGACATGTCTATCTGGCCA

GlaANS2 (431) TTTTTCATTGTATTTACCCTCAAGGGAAACGCGACATGTCTATCTGGCCA

GllANS2 (431) TTTTTCATTGTATTTACCCTCAAGGGAAACGCGACATGTCTATCTGGCCA

Consensus (451) TTTTTCATTGTATTTACCCTCAAGGGAAACGCGACATGTCTATCTGGCCA

501 550

GtANS (501) AAGACTCCCCATGATTACATACCAGCAACAATTGAGTATGCAAAGCAATT

GllANS1 (481) AAGACTCCCCATGATTACATACCGGCAACAATTGAGTATGCAAAGCAATT

GlaANS1 (481) AAGACTCCCCATGATTACATACCGGCAACAATTGAGTATGCAAAGCAATT

GlaANS2 (481) AAGACTCCCCATGATTACATACCGGCAACAATTGAGTATGCAAAGCAATT

GllANS2 (481) AAGACTCCCCATGATTACATACCGGCAACAATTGAGTATGCAAAGCAATT

Consensus (501) AAGACTCCCCATGATTACATACCGGCAACAATTGAGTATGCAAAGCAATT

551 600

GtANS (551) GAGAGACCTAGCTACCAAAGTTCTTGCTGTGCTCTCTGTTGGGCTTGGTT

GllANS1 (531) GAGAGACCTAGCTAGCAAAGTTCTTGCTGTGCTCTCTGTTGGGCTTGGTT

GlaANS1 (531) GAGAGACCTAGCTAGCAAAGTTCTTGCTGTGCTCTCTGTTGGGCTTGGTT

GlaANS2 (531) GAGAGACCTAGCTAGCAAAGTTCTTGCTGTGCTCTCTGTTGGGCTTGGTT

GllANS2 (531) GAGAGACCTAGCTAGCAAAGTTCTTGCTGTGCTCTCTGTTGGGCTTGGTT

Consensus (551) GAGAGACCTAGCTAGCAAAGTTCTTGCTGTGCTCTCTGTTGGGCTTGGTT

601 650

GtANS (601) TAGAACCAGATAGACTTGAAAATGAAGTAGGTGGCATGGAAGAGATGATT

GllANS1 (581) TAGAAGCAGATAGACTTGAAAAGGAAGTAGGTGGCAAGGAAGAGTTAATT

GlaANS1 (581) TAGAAGCAGATAGACTTGAAAAGGAAGTAGGTGGCAAGGAAGAGTTAATT

GlaANS2 (581) TAGAAGCAGATAGACTTGAAAAGGAAGTAGGTGGCAAGGAAGAGTTAATT

GllANS2 (581) TAGAAGCAGATAGACTTGAAAAGGAAGTAGGTGGCAAGGAAGAGTTAATT

Consensus (601) TAGAAGCAGATAGACTTGAAAAGGAAGTAGGTGGCAAGGAAGAGTTAATT

651 700

GtANS (651) CTGCAAAAGAAGATCAATTACTACCCAAAATGCCCTCAACCGGAACTCGC

GllANS1 (631) CTGCAAAAGAAGATCAATTACTACCCAAAGTGTCCTCAACCGGAACTCGC

GlaANS1 (631) CTGCAAAAGAAGATCAATTACTACCCAAAGTGTCCTCAACCGGAACTCGC

GlaANS2 (631) CTGCAAAAGAAGATCAATTACTACCCAAAGTGTCCTCAACCGGAACTCGC

GllANS2 (631) CTGCAAAAGAAGATCAATTACTACCCAAAGTGTCCTCAACCGGAACTCGC

Consensus (651) CTGCAAAAGAAGATCAATTACTACCCAAAGTGTCCTCAACCGGAACTCGC

701 750

GtANS (701) CCTCGGCGTCGAGGCACATACTGATGTCAGTGCTCTTACTTTTATCCTCC

GllANS1 (681) ACTCGGTGTTGAGGCTCACACTGATGTTAGTGCTCTTACTTTTATCCTCC

GlaANS1 (681) ACTCGGTGTTGAGGCTCACACTGATGTTAGTGCTCTTACTTTTATCCTCC

GlaANS2 (681) ACTCGGTGTTGAGGCTCACACTGATGTTAGTGCTCTTACTTTTATCCTCC

GllANS2 (681) GCTCGGGGTTGAGGCTCACACTGATGTTAGTGCTCTTACTTTTATCCTCC

Consensus (701) ACTCGGTGTTGAGGCTCACACTGATGTTAGTGCTCTTACTTTTATCCTCC

751 800

GtANS (751) ACAATATGGTCCCTGGGTTGCAATTGTTTTATCAAGGCAAATGGATCACT

GllANS1 (731) ATAATATGGTTCCTGGCTTACAATTGTTTTATCAAGACAAATGGATCACA

GlaANS1 (731) ATAATATGGTTCCTGGCTTACAATTGTTTTATCAAGACAAATGGATCACA

GlaANS2 (731) ATAATATGGTTCCTGGCTTACAATTGTTTTATCAAGACAAATGGATCACA

GllANS2 (731) ATAATATGGTTCCTGGCTTACAATTGTTTTATCAAGACAAATGGATCACA

Consensus (751) ATAATATGGTTCCTGGCTTACAATTGTTTTATCAAGACAAATGGATCACA

801 850

GtANS (801) GCAAAATGTGTCCCTGATTCCATTATCATGCATGTTGGTGACACACTTGA

GllANS1 (781) GCAAAATGTGTCCCTGATTCCATTATCATGCATGTTGGTGACACACTTGA

GlaANS1 (781) GCAAAATGTGTCCCTGATTCCATTATCATGCATGTTGGTGACACACTTGA

GlaANS2 (781) GCAAAATGTGTCCCTGATTCCATTATCATGCATGTTGGTGACACACTTGA

GllANS2 (781) GCAAAATGTGTCCCTGATTCCATTATCATGCATGTTGGTGACACACTTGA

Consensus (801) GCAAAATGTGTCCCTGATTCCATTATCATGCATGTTGGTGACACACTTGA

851 900

GtANS (851) GATTCTTAGCAATGGCAAGTACAAAAGCATTCTTCATAGAGGGCTTGTCA

GllANS1 (831) GATTCTAAGCAATGGCAAGTACAAAAGCATTCTTCATAGAGGGCTTGTAA

GlaANS1 (831) GATTCTAAGCAATGGCAAGTACAAAAGCATTCTTCATAGAGGGCTTGTAA

GlaANS2 (831) GATTCTAAGCAATGGCAAGTACAAAAGCATTCTTCATAGAGGGCTTGTAA

GllANS2 (831) GATTCTAAGCAATGGCAAGTACAAAAGCATTCTTCATAGAGGGCTTGTGA

Consensus (851) GATTCTAAGCAATGGCAAGTACAAAAGCATTCTTCATAGAGGGCTTGTAA

901 950

GtANS (901) ACAAAGAAAAAGTAAGGATTTCTTGGGCAGTATTTTGTGAGCCACCAAAG

GllANS1 (881) ACAAAGAAAAAGTAAGGATTTCTTGGGCAGTCTTTTGTGAACCACCAAAG

GlaANS1 (881) ACAAAGAAAAAGTAAGGATTTCTTGGGCAGTCTTTTGTGAACCACCAAAG

GlaANS2 (881) ACAAAGAAAAAGTAAGGATTTCTTGGGCAGTCTTTTGTGAACCACCAAAG

GllANS2 (881) ACAAAGAAAAAGTAAGGATTTCTTGGGCAGTCTTTTGTGAACCACCAAAG

Consensus (901) ACAAAGAAAAAGTAAGGATTTCTTGGGCAGTCTTTTGTGAACCACCAAAG

951 1000

GtANS (951) GATAAGATCATTCTTAAGCCACTACCTGAGACAGTATCTGAAATCGAGCC

GllANS1 (931) GATAAAATCATTCTTAAGCCACTCCCTGAGACTGTATCTGAAATCGAACC

GlaANS1 (931) GATAAGATCATTCTTAAGCCACTCCCTGAGACTGTATCTGAAATCGAACC

GlaANS2 (931) GATAAGATCATTCTTAAGCCACTCCCTGAGACTGTATCTGAAATCGAACC

GllANS2 (931) GATAAGATCATTCTTAAGCCACTCCCTGAGACTGTATCTGAAATCGAACC

Consensus (951) GATAAGATCATTCTTAAGCCACTCCCTGAGACTGTATCTGAAATCGAACC

1001 1050

GtANS (1001) GGCTCGATTCCCTCCTCGAACCTTTGCCGAGCATATCAAGCACAAGATCT

GllANS1 (981) GGCTCGATTCCCTCCTCGAACCTTTGCTGAGCATATCAAGCACAAGATCT

GlaANS1 (981) GGCTCGATTCCCTCCTCGAACCTTTGCTGAGCATATCAAGCACAAGATCT

GlaANS2 (981) GGCTCGATTCCCTCCTCGAACCTTTGCTGAGCATATCAAGCACAAGATCT

GllANS2 (981) GGCTCGATTCCCTCCTCGAACCTTTGCTGAGCATATCAAGCACAAGATCT

Consensus (1001) GGCTCGATTCCCTCCTCGAACCTTTGCTGAGCATATCAAGCACAAGATCT

1051 1100

GtANS (1051) TCAGGAAAACTGAAGAGGCTATTAAAGACAACAATATTGCTAATGGCAAT

GllANS1 (1031) TCAGGAAAACTGAAGAAGCTGTTAAAGACAACAATATTGCTAATGGCAAT

GlaANS1 (1031) TCAGGAAAACTGAAGAAGCTGTTAAAGACAACAATATTGCTAATGGCAAT

GlaANS2 (1031) TCAGGAAAACTGAAGAAGCTGTTAAAGACAACAATATTGCTAATGGCAAT

GllANS2 (1031) TCAGGAAAACTGAAGAAGCTGTTAAAGACAACAATATTGCTAATGGCAAT

Consensus (1051) TCAGGAAAACTGAAGAAGCTGTTAAAGACAACAATATTGCTAATGGCAAT

1101 1150

GtANS (1101) **TAG**GACTATTATTTGAATAATCCTCAAGTCTATTTCATGCCGTTAATCAT

GllANS1 (1081) **TAG**

GlaANS1 (1081) **TAG**

GlaANS2 (1081) **TAG**

GllANS2 (1081) **TAG**

Consensus (1101) TAG

**F**

901 950

Gt3GT (901) ATGGCCGCACTCGCGTCAACACTCGAATCAAGAAAGATCCCATTTCTTTG

Gla3GT (1) ATGGCCGCACTCGCGTCAACACTCGAATCACGAAAGATCCCATTTCTTTG

Consensus (901) ATGGCCGCACTCGCGTCAACACTCGAATCA GAAAGATCCCATTTCTTTG

951 1000

Gt3GT (951) GTCTTTGAGAGACGAAGCAAGGAAGCATTTGCCCGAGAATTTCATTGATC

Gla3GT (51) GTCTTTGAGAGATGAAGCAAGGAAGCATTTGCCCGAGAATTTCATTGATC

Consensus (951) GTCTTTGAGAGA GAAGCAAGGAAGCATTTGCCCGAGAATTTCATTGATC

1001 1050

Gt3GT (1001) GAACAAGCACGTTTGGCAAGATTGTTTCTTGGGCACCCCAGTTGCATGTT

Gla3GT (101) GAACAAGCACGTTTGGCAAGATTGTTTCTTGGGCACCCCAGTTGCATGTT

Consensus (1001) GAACAAGCACGTTTGGCAAGATTGTTTCTTGGGCACCCCAGTTGCATGTT

1051 1100

Gt3GT (1051) CTAGAAAACCCCGCTATAGGAGTTTTTGTAACACATTGTGGATGGAATTC

Gla3GT (151) CTAGAAAACCCTGCTATAGGAGTTTTTGTAACACATTGTGGATGGAATTC

Consensus (1051) CTAGAAAACCC GCTATAGGAGTTTTTGTAACACATTGTGGATGGAATTC

1101 1150

Gt3GT (1101) TACCTTGGAGAGTATCTTTTGCCGGGTACCGGTGATCGGTCGGCCGTTTT

Gla3GT (201) TATCTTGGAGAGTATCTTTGCCGGGGTAACAGTGATCGGTCGGCCGTTTT

Consensus (1101) TA CTTGGAGAGTATCTTT C GGGTA C GTGATCGGTCGGCCGTTTT

1151 1200

Gt3GT (1151) TCGGTGATCAGAAAGTGAATGCTAGGATGGTTGAAGATGTTTGGAAAATT

Gla3GT (251) TCGGTGATCAGAAAGTGAATAATAGGATGGTTGAAGATGTTTGGAGAATT

Consensus (1151) TCGGTGATCAGAAAGTGAAT TAGGATGGTTGAAGATGTTTGGA AATT

1201 1250

Gt3GT (1201) GGGGTTGGGGTGAAAGGTGGAGTATTCACTGAAGATGAAACAACTCGTGT

Gla3GT (301) GGGGTTGGGGTGAAAGGTGGAGTATTCACTGAAGATGAAACAGCTCGTGT

Consensus (1201) GGGGTTGGGGTGAAAGGTGGAGTATTCACTGAAGATGAAACA CTCGTGT

1251 1300

Gt3GT (1251) CTTGGAGCTGGTTTTGTTTAGTGACAAAGGTAAAGAAATGAGACAAAATG

Gla3GT (351) CTTGGATCTGGTTTTGTTTAGTGACAAAGGTAAAGAAATGAGAAAAAATG

Consensus (1251) CTTGGA CTGGTTTTGTTTAGTGACAAAGGTAAAGAAATGAGA AAAATG

1301 1350

Gt3GT (1301) TTGGAAGGCTTAAAGAGAAAGCTAAGGATGCTGTAAAAGCAAATGGGAGC

Gla3GT (401) TTGGAAGGCTTAAAGAGAAAGCTAAGGATGCTGTAAAAGCAAATGGGAGC

Consensus (1301) TTGGAAGGCTTAAAGAGAAAGCTAAGGATGCTGTAAAAGCAAATGGGAGC

1351 1400

Gt3GT (1351) TCAACTAGGAATTTTGAGTCTCTATTAGCAGCTTTTAACAAGCTTGATAG

Gla3GT (451) TCAACTAGGAATTTCGAGTCTCTATTAGCAGCTTTTAAC**TAG**CCTGGTAG

Consensus (1351) TCAACTAGGAATTT GAGTCTCTATTAGCAGCTTTTAAC AGC TG TAG

1401 1450

Gt3GT (1401) TTAGTTAATAC**TAG**CAATGTAATAGTACGTTATACAGCAACTTTATGGTA

Gla3GT (501) ----TTAATACTAGCAATGTAATAGTACGTTATACAGCAA

Consensus (1401) TTAATACTAGCAATGTAATAGTACGTTATACAGCAA

**G**

901 950

GtF3´H (901) TAATATCGACGGTGGTGACGAAGGAACCAAACTCACAGATACTGAAATCA

GlaF3´H (1) GAAGGAACCAAACTCACAGATACTGAAATAA

Consensus (901) GAAGGAACCAAACTCACAGATACTGAAAT A

951 1000

GtF3´H (951) AAGCTCTCCTTTTGAACTTGTTCATAGCCGGAACAGACACTTCATCAAGT

GlaF3´H (32) AAGCTCTCCTTTTGAACTTGTTCATAGCTGGAACAGACACTTCATCAAGT

Consensus (951) AAGCTCTCCTTTTGAACTTGTTCATAGC GGAACAGACACTTCATCAAGT

1001 1050

GtF3´H (1001) ACTGTAGAATGGGCCATGGCAGAACTAATCCGAAACCCAAAGTTACTAGT

GlaF3´H (82) ACTGTAGAATGGGCCATTGCAGAACTAATCCGAAACCCAAAACTACTTGT

Consensus (1001) ACTGTAGAATGGGCCAT GCAGAACTAATCCGAAACCCAAA TACT GT

1051 1100

GtF3´H (1051) CCAAGCCCAAGAAGAGCTAGACAGAGTAGTCGGGCCGAACCGATTCGTAA

GlaF3´H (132) CCAAGCCCAAGAAGAGCTGGACAGAGTTGTCGGGCCGAATCGATTAGTTA

Consensus (1051) CCAAGCCCAAGAAGAGCT GACAGAGT GTCGGGCCGAA CGATT GT A

1101 1150

GtF3´H (1101) CCGAATCTGATCTTCCTCAACTGACATTCCTTCAAGCCGTCATCAAAGAG

GlaF3´H (182) CCGAATCCGATCTTCCTGAATTGACATTCCTTCAAGCCATCATCAAAGAG

Consensus (1101) CCGAATC GATCTTCCT AA TGACATTCCTTCAAGCC TCATCAAAGAG

1151 1200

GtF3´H (1151) ACTTTCAGGCTTCATCCATCCACCCCACTCTCTCTTCCACGAATGGCGGC

GlaF3´H (232) ACTTTCAGGCTTCATCCATCTACTCCTCTCTCTCTTCCCCGAATGGCGGC

Consensus (1151) ACTTTCAGGCTTCATCCATC AC CC CTCTCTCTTCC CGAATGGCGGC

1201 1250

GtF3´H (1201) GGAGGACTGTGAGATCAATGGGTATTATGTCTCAGAAGGTTCGACATTGC

GlaF3´H (282) GGAGGACTGTGAGATCGATGGGTATTATGTCTCAAAAGGTACGACATTGC

Consensus (1201) GGAGGACTGTGAGATC ATGGGTATTATGTCTCA AAGGT CGACATTGC

1251 1300

GtF3´H (1251) TCGTCAATGTGTGGGCCATAGCTCGTGATCCAAATGCGTGGGCCAATCCA

GlaF3´H (332) TCGTTAATGTGTGGGCCATAGCTCGTGATCCAACTATGTGGGCCGATCCA

Consensus (1251) TCGT AATGTGTGGGCCATAGCTCGTGATCCAA T GTGGGCC ATCCA

1301 1350

GtF3´H (1301) CTAGATTTCAACCCGACTCGTTTCTTGGCCGGTGGAGAGAAGCCTAATGT

GlaF3´H (382) TTAGCTTTTAATCCGGCCCGTTTCTTGGCTGGTGGAGAGAAGCCTAATGT

Consensus (1301) TAG TTT AA CCG C CGTTTCTTGGC GGTGGAGAGAAGCCTAATGT

1351 1400

GtF3´H (1351) TGATGTTAAAGGAAATGATTTTGAAGTGATACCTTTCGGTGCTGGGCGCA

GlaF3´H (432) TGATGTTAAAGGAAATGATTTTGAGGTGATACCGTTTGGTGCTGGGCGTA

Consensus (1351) TGATGTTAAAGGAAATGATTTTGA GTGATACC TT GGTGCTGGGCG A

1401 1450

GtF3´H (1401) GGATATGTGCCGGAATGAGCTTAGGTATACGGATGGTTCAACTAGTAACG

GlaF3´H (482) GGATATGTGCTGGAATGAGCTTAGGTATACGAATGGTTCAACTAGTAACG

Consensus (1401) GGATATGTGC GGAATGAGCTTAGGTATACG ATGGTTCAACTAGTAACG

1451 1500

GtF3´H (1451) GCTTCGTTAGTTCATTCGTTTGATTGGGCTTTGTTGGATGGACTTAAACC

GlaF3´H (532) GCTTCGTTAGTTCAGTCGTTTGATTGGGCTTTGTTGCACGGACTTAAACC

Consensus (1451) GCTTCGTTAGTTCA TCGTTTGATTGGGCTTTGTTG A GGACTTAAACC

1501 1550

GtF3´H (1501) CGAGAAGCTTGACATGGAGGAAGGTTATGGACTAACGCTTCAACGAGCTT

GlaF3´H (582) CGAGAAGCTTGACATGGAGGAAGGTTACGGACTAACGCTTCAACGAGCTT

Consensus (1501) CGAGAAGCTTGACATGGAGGAAGGTTA GGACTAACGCTTCAACGAGCTT

1551 1600

GtF3´H (1551) CACCTTTAATCGTCCATCCAAAGCCGAGGCTCTCGGCTCAAGTTTATTGT

GlaF3´H (632) CACCTTT

Consensus (1551) CACCTTT

1601 1650

GtF3´H (1601) ATG**TAA**CAAGTTTGTGAAGCCAGTCTGATTTCAGTTGGATTTGTAGTTAT

GlaF3´H (639)

Consensus (1601)

**H**

901 950

GtF3´5´H (901) TCAAGGCTCTTTTATTGAACTTGTTTACTGCTGGTACGGATACATCATCA

GlaF3´5´H (1) TACTGCTGGTACGGATACATCATCA

Consensus (901) TACTGCTGGTACGGATACATCATCA

951 1000

GtF3´5´H (951) AGCATCATTGAGTGGGCACTAGCAGAACTGCTAAAGAATCGGACACTCCT

GlaF3´5´H (26) AGCATCATAGAGTGGGCACTAGCAGAGCTGCTAAAGAATCCAACCCTCCT

Consensus (951) AGCATCAT GAGTGGGCACTAGCAGA CTGCTAAAGAATC AC CTCCT

1001 1050

GtF3´5´H (1001) CACCCGAGCCCAGGACGAAATGGATCGGGTAATCGGGCGAGACCGCCGTC

GlaF3´5´H (76) GACCCGAGCCCAGGACGAAATGGATCGGGTAATCGGTCGAGACCGCCGTC

Consensus (1001) ACCCGAGCCCAGGACGAAATGGATCGGGTAATCGG CGAGACCGCCGTC

1051 1100

GtF3´5´H (1051) TTCTTGAATCAGACATCCCCAACTTACCATATCTTCAAGCAATCTGCAAA

GlaF3´5´H (126) TTCTCGAATCAGACATCCCCAAATTACCATACCTTGAAGCAATCTGCAAA

Consensus (1051) TTCT GAATCAGACATCCCCAA TTACCATA CTT AAGCAATCTGCAAA

1101 1150

GtF3´5´H (1101) GAAACATTCCGTAAACACCCTTCAACACCATTAAACCTTCCAAGGAATTG

GlaF3´5´H (176) GAAACATTCCGCAAACACCCATCAACCCCATTAAACCTTCCAAG-AATTG

Consensus (1101) GAAACATTCCG AAACACCC TCAAC CCATTAAACCTTCCAAG AATTG

1151 1200

GtF3´5´H (1151) CATCAGAGGCCATGTGGATGTAAATGGGTACTACATTCCGAAAGGGACTC

GlaF3´5´H (225) CATCAGAA-CCATGTG-AAGTAAATGGGTACTACATTCCGAAAGGAACTC

Consensus (1151) CATCAGA CCATGTG A GTAAATGGGTACTACATTCCGAAAGG ACTC

1201 1250

GtF3´5´H (1201) GGCTCAACGTCAACATATGGGCGATTGGAAGAGACCCATCGGTTTGGGGG

GlaF3´5´H (273) GGCTCAACGTCAACATATGGGCAATTGGAAGAGACCCATCTGTATG---G

Consensus (1201) GGCTCAACGTCAACATATGGGC ATTGGAAGAGACCCATC GT TG G

1251 1300

GtF3´5´H (1251) GATAACCCGAACGAGTTCGACCCGGAGAGGTTTTTGTATGGGAGGAATGC

GlaF3´5´H (320) GATAACCCGAATGAGTTCGACCCGGAAAGATTTTTGTATGGGAAGAATGC

Consensus (1251) GATAACCCGAA GAGTTCGACCCGGA AG TTTTTGTATGGGA GAATGC

1301 1350

GtF3´5´H (1301) TAAGATTGATCCACGAGGAAACCATTTTGAATTGATCCCATTTGGTGCTG

GlaF3´5´H (370) TAAGATTGATCCACGAGGAAACGATTTTGAATTGATCCCATTTGGAGCTG

Consensus (1301) TAAGATTGATCCACGAGGAAAC ATTTTGAATTGATCCCATTTGG GCTG

1351 1400

GtF3´5´H (1351) GACGAAGAATTTGTGCAGGAACAAGAATGGGGATATTGCTTGTTGAGTAT

GlaF3´5´H (420) GACGAAGAATTTGCGCTGGAACAAGAATGGGGATATTGCTTGTTGAGTAT

Consensus (1351) GACGAAGAATTTG GC GGAACAAGAATGGGGATATTGCTTGTTGAGTAT

1401 1450

GtF3´5´H (1401) ATTTTGGGGACATTGGTGCATAGTTTTGATTGGAAACTGGGATTTTCTGA

GlaF3´5´H (470) ATTTTGGGGACATTGCTGCATTCTTTTGATTGGAAATTGGAGTTTTCTGA

Consensus (1401) ATTTTGGGGACATTG TGCAT TTTTGATTGGAAA TGG TTTTCTGA

1451 1500

GtF3´5´H (1451) GGATGAGCTTAATATGGATGAGACATTTGGGCTTGCTCTGCAGAAAGCTG

GlaF3´5´H (520) GGATGAGCTTAATATGGATGAGACATTTGGGCTTGCTCTGCAG

Consensus (1451) GGATGAGCTTAATATGGATGAGACATTTGGGCTTGCTCTGCAG

1501 1550

GtF3´5´H (1501) TGCCTTTAGCGGCCATGGTTATTCCACGCCTTCCTCTTCATGTTTATGCT

GlaF3´5´H (563)

Consensus (1501)

1551 1600

GtF3´5´H (1551) CCT**TAA**TTCAGAGATTTAATTTCATGCTTTGTTTTATTAATCATTTTCTT

GlaF3´5´H (563)

Consensus (1551)

**S4 Fig.** **Alignments of cDNA sequences encoded anthocyanin biosynthetic enzymes among *Gentiana triflora, G. lutea* L. var. *lutea* and *G. lutea* L. var. *aurantiaca*.** The underlined cDNA sequences are primers used to isolate cDNAs from *lutea* and *aurantiaca*. The start codon (ATG) and stop codons (TGA, TAG or TAA) are underlined with bold letters. Gaps are insered with a dash (-) in one of the sequences. Abbreviations: *triflora*, *Gentiana triflora*; *aurantiaca*, *G. lutea* L. var. *aurantiaca*; *lutea, G. lutea* L. var. *lutea*; Gt, *Gentiana triflora*; Gll, *G. lutea* L. var. *lutea*; Gla, *G. lutea* L. var. *aurantiaca*; CHS, chalcone synthase; CHI, chalcone isomerase; F3H, flavonone 3-hydroxylase; DFR, dihydroflavonol 4-reductase; ANS, anthocyanidin synthase; 3GT, UDP-glucose:flavonoid-3-*O*-glucosyltransferase; F3´H, for flavonoid 3'-hydroxylase; F3´5´H, flavonoid 3',5'-hydroxylase. GenBank accession numbers: GtCHS, D38043; GtCHI, D38168; GtDFR, D85185; GtANS, AB193310; Gt3GT, D85186; GtF3´H, AB193313; GtF3´5´H, D85184; GtF3H1, AB193311; GtF3H2, AB193312. The cDNA sequences encoded anthocyanin biosynthetic enzymes from *lutea* and *aurantiaca* are isolated by authors in this study.
