# Supplementary material for: Red Anthocyanins and Yellow Carotenoids Form the Color of Orange-Flower Gentian (Gentiana lutea L. var. aurantiaca)
Source: PLoS One. 2016 Sep 2;11(9):e0162410. doi: 10.1371/journal.pone.0162410 (PMC5010251; doi:10.1371/journal.pone.0162410)
Supplement: S5 Fig — The first methionine (M) and stop codon are marked with underlined bold letter and the asterisk, respectively. Gaps are insered with a dash (-) in one of the sequences. The underlined amino acid sequences from lutea and aurantiaca were deduced from primers. Abbreviations: triflora, Gentiana triflora; aurantiaca, G. lutea L. var. aurantiaca; lutea, G. lutea L. var. lutea; Gt, Gentiana triflora; Gll, G. lutea L. var. lutea; Gla, G. lutea L. var. aurantiaca; CHS, chalcone synthase; CHI, chalcone isomerase; ANS, anthocyanidin synthase; 3GT, UDP-glucose:flavonoid-3-O-glucosyltransferase; F3´H, for flavonoid 3'-hydroxylase; F3´5´H, flavonoid 3',5'-hydroxylase. GenBank accession numbers: GtCHS, D38043; GtCHI, D38168; GtANS, AB193310; Gt3GT, D85186; GtF3´H, AB193313; GtF3´5´H, D85184; GtF3H1, AB193311; GtF3H2, AB193312. The cDNA sequences encoded anthocyanin biosynthetic enzymes from lutea and aurantiaca are isolated by authors in this study. (DOC) [file pone.0162410.s005.doc]

**A**

1 50

GtCHS (1) **M**VTVEEIRKAQRAEGPATVLAIGTATPVNCVDQSTYPDYYFRITDSEHKT

GllCHS (1) **M**VTVEEIRNAQRAEGPATVLAIGTATPINCVDQSTYPDYYFRITDSEHKT

GlaCHS (1) **M**VTVEEIRNAQRAEGPATVLAIGTATPINCVDQSTYPDYYFRITDSEHKT

Consensus (1) MVTVEEIRNAQRAEGPATVLAIGTATPINCVDQSTYPDYYFRITDSEHKT

51 100

GtCHS (51) ELKEKFKRMCEKSMIRKRYMHLTEDILKENPNMCAYMAPSLDARQDIVVV

GllCHS (51) ELKEKFKRMCEKSMIRQRYMHLTEDILKENPNICAYMAPSLDARQDIVVV

GlaCHS (51) ELKEKFKRMCEKSMIRQRYMHLTEDILKENPNICAYMAPSLDARQDIVVV

Consensus (51) ELKEKFKRMCEKSMIRQRYMHLTEDILKENPNICAYMAPSLDARQDIVVV

101 150

GtCHS (101) EVPKLGKEAAQKAIKEWGQPKSKITHLVFCTTSGVDMPGADYQITKLLGL

GllCHS (101) EVPKLGKEAAQKAIKEWGQPKSKITHLVVCTTSGVDMPGADYQITKLLGL

GlaCHS (101) EVPKLGKEAAQKAIKEWGQPKSKITHLVVCTTSGVDMPGADYQITKLLGL

Consensus (101) EVPKLGKEAAQKAIKEWGQPKSKITHLVVCTTSGVDMPGADYQITKLLGL

151 200

GtCHS (151) RSSVKRFMMYQQGCFAGGTVLRMAKDLAENNRGARVLVVCSEITAVTFRG

GllCHS (151) RSSVKRFMMYQQGCFAGGTVLRMAKDLAENNRGARVLVVCSEITAVTFRG

GlaCHS (151) RSSVKRFMMYQQGCFAGGTVLRMAKDLAENNRGARVLVVCSEITAVTFRG

Consensus (151) RSSVKRFMMYQQGCFAGGTVLRMAKDLAENNRGARVLVVCSEITAVTFRG

201 250

GtCHS (201) PSESHLDSLVGQALFGDGAAAIIVGSDPIPDLERPLFQIVSAAQTLLPDS

GllCHS (201) PSESHLDSLVGQALFGDGAAAIIVGSDPIPDLERPLFQIVSAAQTLLPDS

GlaCHS (201) PSESHLDSLVGQALFGDGAAAIIVGSDPIPDLERPLFQIVSAAQTLLPDS

Consensus (201) PSESHLDSLVGQALFGDGAAAIIVGSDPIPDLERPLFQIVSAAQTLLPDS

251 300

GtCHS (251) HGAIDGHLREVGLTFHLLKDVPGLISKHIEKSLKEAFDPIGISDWNSIFW

GllCHS (251) HGAIDGHLREVGLTFHLLKDVPGLISKHIQKSLKEAFEPIGISDWNSIFW

GlaCHS (251) HGAIDGHLREVGLTFHLLKDVPGLISKHIQKSLKEAFEPIGISDWNSIFW

Consensus (251) HGAIDGHLREVGLTFHLLKDVPGLISKHIQKSLKEAFEPIGISDWNSIFW

301 350

GtCHS (301) IAHPGGPAILDQVEETLGLEAQKLRATRHVLAEYGNMSSACVLFILDEMR

GllCHS (301) IAHPGGPAILDQVEETLGLEAQKLRSTRHVLSEYGNMSSACVLFILDEMR

GlaCHS (301) IAHPGGPAILDQVEETLGLEAQKLRSTRHVLSEYGNMSSACVLFILDEMR

Consensus (301) IAHPGGPAILDQVEETLGLEAQKLRSTRHVLSEYGNMSSACVLFILDEMR

351 390

GtCHS (351) KTSAKDGATTTGEGQDWGVLFGFGPGLTVETVVLHSVSV*

GllCHS (351) KSSARDGASTTGEGLDWGVLFGFGPGLTVETVVLHSVSV*

GlaCHS (351) KSSARDGASTTGEGLDWGVLFGFGPGLTVETVVLHSVSV*

Consensus (351) KSSARDGASTTGEGLDWGVLFGFGPGLTVETVVLHSVSV*

**B**

1 50

GtCHI (1) **M**VSSSVSSVTEVKVESYVFPPSVKPPSSTKSFLLGGAGVRGLEINGNFVK

GllCHI (1) SVTEVKVESYVFPPSVKPPSSPKSFLLGGAGVRGLDINGNFVK

GlaCHI (1) SVTEVKVESYVFPPSVKPPSSPKSFLLGGAGVRGLDINGNFVK

Consensus (1) SVTEVKVESYVFPPSVKPPSSPKSFLLGGAGVRGLDINGNFVK

51 100

GtCHI (51) FTAIGVYLEESGVAVLSGKWKGKTAEELSDSVEFFTDIITGPFEKFTQVT

GllCHI (44) FTAIGVYLEENGVALLAGKWKGKTAEELTDSVEFFSDIITGPFEKLTHGT

GlaCHI (44) FTAIGVYLEENGVALLAGKWKGKTAEELTDSVEFFSDIITGPFEKLTHGT

Consensus (51) FTAIGVYLEENGVALLAGKWKGKTAEELTDSVEFFSDIITGPFEKLTHGT

101 150

GtCHI (101) LILPVTGQQYSPKVAENCAAQWKAAGIYTDADGIAIEKFLQVFQTESFTP

GllCHI (94) FILPLTGEQYSAKVAENCVAQWKAAGIYTDADGIAIEKFLQVFQTKSFTT

GlaCHI (94) FILPLTGEQYSAKVAENCVAQWKAAGIYTDADGIAIEKFLQVFQTKSFTT

Consensus (101) FILPLTGEQYSAKVAENCVAQWKAAGIYTDADGIAIEKFLQVFQTKSFTT

151 200

GtCHI (151) GDSILFTHSPESLTISFGKNGAI-PEVSNAVIENKKLSEAVIESIIGEKG

GllCHI (144) GDYVLYTHSPESLTISFGKNGAIIPEVGNAVIENKKLSEAVIESIIGEKG

GlaCHI (144) GDYVLYTHSPESLTISFGKNGAIIPEVGNAVIENKKLSEAVIESIIGEKG

Consensus (151) GDYVLYTHSPESLTISFGKNGAIIPEVGNAVIENKKLSEAVIESIIGEKG

201 223

GtCHI (200) VSPAAKKSLATRIAEILNHFDA*

GllCHI (194) VSPAAKKSLATR

GlaCHI (194) VSPAAKKSLATR

Consensus (201) VSPAAKKSLATR

**C**

1 50

GtANS (1) **M**GSLLPSRVESLAISGIKTIPKEYIRPKEELASIGNIFEEAKNNNKTSQI

GllANS1 (1) **M**GSLLPSRVESLAMSGIKTIPKEYVRPKEEVASIGNVFEE----DETQTQ

GlaANS1 (1) **M**GSLLPSRVESLAMSGIKTIPKEYVRPKEEVASIGNVFEE----DETQTQ

GlaANS2 (1) **M**GSLLPSRVESLAMSCIKTIPREYVRPKEEVASIGNVFEE----DETQTQ

GllANS2 (1) **M**GSLLPSRVESLAMSCIKTIPREYVRPKEEVASIGNVFEE----DETQTQ

Consensus (1) MGSLLPSRVESLAMSGIKTIPKEYVRPKEEVASIGNVFEE DETQTQ

51 100

GtANS (51) VPTIDLKDMDSLDNNKDVQTQCHDELKNAAMEWGVMNLVNHGISQELINR

GllANS1 (47) VPTIDLKDLDSLDD-KDVQTRCHDELKKAAMEWGVMHLVNHGISYDIINR

GlaANS1 (47) VPTIDLKDLDSLDD-KDVQTRCHDELKKAAMEWGVMHLVNHGISYDIINR

GlaANS2 (47) VPTIDLKDLDSLDN-KDVQTRCHDELKKAAMEWGVMHLVNHGISYDIINR

GllANS2 (47) VPTIDLKDLDSLDN-KDVQTRCHDELKKAAMEWGVMHLVNHGISYDIINR

Consensus (51) VPTIDLKDLDSLDN KDVQTRCHDELKKAAMEWGVMHLVNHGISYDIINR

101 150

GtANS (101) VKSAGQAFFDLPIEEKEKYANDQASGNVQGYGSRLANNASGQLEWEDYFF

GllANS1 (96) VKSAGQAFFDLPIEEKEKYANDQASGNVQGYGSRLANNASGQLEWEDYFF

GlaANS1 (96) VKSAGQAFFDLPIEEKEKYANDQASGNVQGYGSRLANNASGQLEWEDYFF

GlaANS2 (96) VKSAGQAFFDLPIEEKEKYANDQASGNVQGYGSRLANNASGQLEWEDYFF

GllANS2 (96) VKSAGQAFFDLPIEEKEKYANDQASGNVQGYGSRLANNASGQLEWEDYFF

Consensus (101) VKSAGQAFFDLPIEEKEKYANDQASGNVQGYGSRLANNASGQLEWEDYFF

151 200

GtANS (151) HCIYPERKRDMSIWPKTPHDYIPATIEYAKQLRDLATKVLAVLSVGLGLE

GllANS1 (146) HCIYPQGKRDMSIWPKTPHDYIPATIEYAKQLRDLASKVLAVLSVGLGLE

GlaANS1 (146) HCIYPQGKRDMSIWPKTPHDYIPATIEYAKQLRDLASKVLAVLSVGLGLE

GlaANS2 (146) HCIYPQGKRDMSIWPKTPHDYIPATIEYAKQLRDLASKVLAVLSVGLGLE

GllANS2 (146) HCIYPQGKRDMSIWPKTPHDYIPATIEYAKQLRDLASKVLAVLSVGLGLE

Consensus (151) HCIYPQGKRDMSIWPKTPHDYIPATIEYAKQLRDLASKVLAVLSVGLGLE

201 250

GtANS (201) PDRLENEVGGMEEMILQKKINYYPKCPQPELALGVEAHTDVSALTFILHN

GllANS1 (196) ADRLEKEVGGKEELILQKKINYYPKCPQPELALGVEAHTDVSALTFILHN

GlaANS1 (196) ADRLEKEVGGKEELILQKKINYYPKCPQPELALGVEAHTDVSALTFILHN

GlaANS2 (196) ADRLEKEVGGKEELILQKKINYYPKCPQPELALGVEAHTDVSALTFILHN

GllANS2 (196) ADRLEKEVGGKEELILQKKINYYPKCPQPELALGVEAHTDVSALTFILHN

Consensus (201) ADRLEKEVGGKEELILQKKINYYPKCPQPELALGVEAHTDVSALTFILHN

251 300

GtANS (251) MVPGLQLFYQGKWITAKCVPDSIIMHVGDTLEILSNGKYKSILHRGLVNK

GllANS1 (246) MVPGLQLFYQDKWITAKCVPDSIIMHVGDTLEILSNGKYKSILHRGLVNK

GlaANS1 (246) MVPGLQLFYQDKWITAKCVPDSIIMHVGDTLEILSNGKYKSILHRGLVNK

GlaANS2 (246) MVPGLQLFYQDKWITAKCVPDSIIMHVGDTLEILSNGKYKSILHRGLVNK

GllANS2 (246) MVPGLQLFYQDKWITAKCVPDSIIMHVGDTLEILSNGKYKSILHRGLVNK

Consensus (251) MVPGLQLFYQDKWITAKCVPDSIIMHVGDTLEILSNGKYKSILHRGLVNK

301 350

GtANS (301) EKVRISWAVFCEPPKDKIILKPLPETVSEIEPARFPPRTFAEHIKHKIFR

GllANS1 (296) EKVRISWAVFCEPPKDKIILKPLPETVSEIEPARFPPRTFAEHIKHKIFR

GlaANS1 (296) EKVRISWAVFCEPPKDKIILKPLPETVSEIEPARFPPRTFAEHIKHKIFR

GlaANS2 (296) EKVRISWAVFCEPPKDKIILKPLPETVSEIEPARFPPRTFAEHIKHKIFR

GllANS2 (296) EKVRISWAVFCEPPKDKIILKPLPETVSEIEPARFPPRTFAEHIKHKIFR

Consensus (301) EKVRISWAVFCEPPKDKIILKPLPETVSEIEPARFPPRTFAEHIKHKIFR

351 366

GtANS (351) KTEEAIKDNNIANGN*

GllANS1 (346) KTEEAVKDNNIANGN*

GlaANS1 (346) KTEEAVKDNNIANGN*

GlaANS2 (346) KTEEAVKDNNIANGN*

GllANS2 (346) KTEEAVKDNNIANGN*

Consensus (351) KTEEAVKDNNIANGN*

**D**

251 300

Gt3GT (251) SIPPEDNECLKWLQTQKESSVVYLSFGTVINPPPNEMAALASTLESRKIP

Gla3GT (1) MAALASTLESRKIP

Consensus (251) MAALASTLESRKIP

301 350

Gt3GT (301) FLWSLRDEARKHLPENFIDRTSTFGKIVSWAPQLHVLENPAIGVFVTHCG

Gla3GT (15) FLWSLRDEARKHLPENFIDRTSTFGKIVSWAPQLHVLENPAIGVFVTHCG

Consensus (301) FLWSLRDEARKHLPENFIDRTSTFGKIVSWAPQLHVLENPAIGVFVTHCG

351 400

Gt3GT (351) WNSTLESIFCRVPVIGRPFFGDQKVNARMVEDVWKIGVGVKGGVFTEDET

Gla3GT (65) WNSILESIFAGVTVIGRPFFGDQKVNNRMVEDVWRIGVGVKGGVFTEDET

Consensus (351) WNS LESIF V VIGRPFFGDQKVN RMVEDVWKIGVGVKGGVFTEDET

401 450

Gt3GT (401) TRVLELVLFSDKGKEMRQNVGRLKEKAKDAVKANGSSTRNFESLLAAFNK

Gla3GT (115) ARVLDLVLFSDKGKEMRKNVGRLKEKAKDAVKANGSSTRNFESLLAAFN-

Consensus (401) RVLDLVLFSDKGKEMR NVGRLKEKAKDAVKANGSSTRNFESLLAAFN

451

Gt3GT (451) LDS*

Gla3GT (164) ---*

Consensus (451)

**E**

251 300

GtF3´H (251) LHARFDKFLNGILEDRKSNGSNGAEQYVDLLSVLISLQDSNIDGGDEGTK

GlaF3´H (1) EGTK

Consensus (251) EGTK

301 350

GtF3´H (301) LTDTEIKALLLNLFIAGTDTSSSTVEWAMAELIRNPKLLVQAQEELDRVV

GlaF3´H (5) LTDTEIKALLLNLFIAGTDTSSSTVEWAIAELIRNPKLLVQAQEELDRVV

Consensus (301) LTDTEIKALLLNLFIAGTDTSSSTVEWAIAELIRNPKLLVQAQEELDRVV

351 400

GtF3´H (351) GPNRFVTESDLPQLTFLQAVIKETFRLHPSTPLSLPRMAAEDCEINGYYV

GlaF3´H (55) GPNRLVTESDLPELTFLQAIIKETFRLHPSTPLSLPRMAAEDCEIDGYYV

Consensus (351) GPNR VTESDLP LTFLQAIIKETFRLHPSTPLSLPRMAAEDCEI GYYV

401 450

GtF3´H (401) SEGSTLLVNVWAIARDPNAWANPLDFNPTRFLAGGEKPNVDVKGNDFEVI

GlaF3´H (105) SKGTTLLVNVWAIARDPTMWADPLAFNPARFLAGGEKPNVDVKGNDFEVI

Consensus (401) S GSTLLVNVWAIARDP WA PL FNP RFLAGGEKPNVDVKGNDFEVI

451 500

GtF3´H (451) PFGAGRRICAGMSLGIRMVQLVTASLVHSFDWALLDGLKPEKLDMEEGYG

GlaF3´H (155) PFGAGRRICAGMSLGIRMVQLVTASLVQSFDWALLHGLKPEKLDMEEGYG

Consensus (451) PFGAGRRICAGMSLGIRMVQLVTASLV SFDWALL GLKPEKLDMEEGYG

501 525

GtF3´H (501) LTLQRASPLIVHPKPRLSAQVYCM*

GlaF3´H (205) LTLQRASP

Consensus (501) LTLQRASP

**F**

301 350

GtF3´5´H (301) ALLLNLFTAGTDTSSSIIEWALAELLKNRTLLTRAQDEMDRVIGRDRRLL

GlaF3´5´H (1) TAGTDTSSSIIEWALAELLKNPTLLTRAQDEMDRVIGRDRRLL

Consensus (301) TAGTDTSSSIIEWALAELLKN TLLTRAQDEMDRVIGRDRRLL

351 400

GtF3´5´H (351) ESDIPNLPYLQAICKETFRKHPSTPLNLPRNCIRGHVDVNGYYIPKGTRL

GlaF3´5´H (44) ESDIPKLPYLEAICKETFRKHPSTPLNLPR-IASEPCEVNGYYIPKGTRL

Consensus (351) ESDIP LPYL AICKETFRKHPSTPLNLPR DVNGYYIPKGTRL

401 450

GtF3´5´H (401) NVNIWAIGRDPSVWGDNPNEFDPERFLYGRNAKIDPRGNHFELIPFGAGR

GlaF3´5´H (93) NVNIWAIGRDPSVW-DNPNEFDPERFLYGKNAKIDPRGNDFELIPFGAGR

Consensus (401) NVNIWAIGRDPSVW DNPNEFDPERFLYGKNAKIDPRGN FELIPFGAGR

451 500

GtF3´5´H (451) RICAGTRMGILLVEYILGTLVHSFDWKLGFSEDELNMDETFGLALQKAVP

GlaF3´5´H (142) RICAGTRMGILLVEYILGTLLHSFDWKLEFSEDELNMDETFGLALQ

Consensus (451) RICAGTRMGILLVEYILGTLLHSFDWKL FSEDELNMDETFGLALQ

501 517

GtF3´5´H (501) LAAMVIPRLPLHVYAP*

GlaF3´5´H (188)

Consensus (501)

**S5 Fig.** **Alignments of the deduced amino acid sequences encoded by anthocyanin biosynthetic genes from *Gentiana triflora, G. lutea* L. var. *lutea*, and *G. lutea* L. var. *aurantiaca*.** The first methionine (M) and stop codon are marked with underlined bold letter and the asterisk, respectively. Gaps are insered with a dash (-) in one of the sequences. The underlined amino acid sequences from *lutea* and *aurantiaca* were deduced from primers. Abbreviations: *triflora*, *Gentiana triflora*; *aurantiaca*, *G. lutea* L. var. *aurantiaca*; *lutea, G. lutea* L. var. *lutea*; Gt, *Gentiana triflora*; Gll, *G. lutea* L. var. *lutea*; Gla, *G. lutea* L. var. *aurantiaca*; CHS, chalcone synthase; CHI, chalcone isomerase; ANS, anthocyanidin synthase; 3GT, UDP-glucose:flavonoid-3-*O*-glucosyltransferase; F3´H, for flavonoid 3'-hydroxylase; F3´5´H, flavonoid 3',5'-hydroxylase. GenBank accession numbers: GtCHS, D38043; GtCHI, D38168; GtANS, AB193310; Gt3GT, D85186; GtF3´H, AB193313; GtF3´5´H, D85184; GtF3H1, AB193311; GtF3H2, AB193312. The cDNA sequences encoded anthocyanin biosynthetic enzymes from *lutea* and *aurantiaca* are isolated by authors in this study.
