# Supplementary material for: Red Anthocyanins and Yellow Carotenoids Form the Color of Orange-Flower Gentian (Gentiana lutea L. var. aurantiaca)
Source: PLoS One. 2016 Sep 2;11(9):e0162410. doi: 10.1371/journal.pone.0162410 (PMC5010251; doi:10.1371/journal.pone.0162410)
Supplement: S1 Table — Abbreviations: CHS, chalcone synthase; CHI, chalcone isomerase; F3H, flavonone 3-hydroxylase; DFR, dihydroflavonol 4-reductase; ANS, anthocyanidin synthase; 3GT, UDP-glucose:flavonoid-3-O-glucosyltransferase; F3'H, flavonoid 3'-hydroxlase; F3'5'H, flavonoid 3',5'-hydroxylase. (DOC) [file pone.0162410.s006.doc]

**S1 Table.** **Oligonucleotide sequences of primer pairs used for RT-PCR**.

| Genes | Direction | Sequence (5´→ 3´) | Accession numbers | References |
| --- | --- | --- | --- | --- |
| *CHS* | Forward | GATCAAGAATGGTGACCGTTGAGGAGATCAGA | D38043 | [21] |
|  | Reverse | CTCTCTCAAACCGAAACGCTGTGTAACACGAC |  |  |
| *CHI* | Forward | TCCGTCACCGAAGTTAAAGTCGAGAGCTACGTT | D38168 | [23] |
|  | Reverse | TTCTTGTTGCCAAGCTCTTCTTTGCAGCT |  |  |
| *F3H* | Forward | GTTTGATATGTCCGGTGGTAAGAAAGGTGGTT | AB193311 & AB193312 | [23] |
|  | Reverse | CATTAAATATTATAAAATATATAATGCGATGC |  |  |
| *DFR* | Forward | ATGGAAGGAGGGATTTTATCAAATGCCACAA | D85185 | [20] |
|  | Reverse | TCTAGTCTAGTGAATCTTGAATCAGTTCCAT |  |  |
| *ANS* | Forward | ATGGGATCTCTTTTGCCTAGTAGAGTTGA | AB193310 | [23] |
|  | Reverse | CTAATTGCCATTAGCAATATTGTTGTCTT |  |  |
| *3GT* | Forward | ATGGCCGCACTCGCGTCAACACTCGAATCA | AB076697 | [6] |
|  | Reverse | TTGCTGTATAACGTACTATTACATTGCTAG |  |  |
| *F3´H* | Forward | GAAGGAACCAAACTCACAGATACTGAAA | AB193313 | [23] |
|  | Reverse | AAAGGTGAAGCTCGTTGAAGCGTTAGT |  |  |
| *F3´5´H* | Forward | TACTGCTGGTACGGATACATCATCAAG | D85184 | [20] |
|  | Reverse | CTGCAGAGCAAGCCCAAATGTCTCATC |  |  |

Abbreviations: CHS, chalcone synthase; CHI, chalcone isomerase; F3H, flavonone 3-hydroxylase; DFR, dihydroflavonol 4-reductase; ANS, anthocyanidin synthase; 3GT, UDP-glucose:flavonoid-3-*O*-glucosyltransferase; F3'H, flavonoid 3'-hydroxlase; F3'5'H, flavonoid 3',5'-hydroxylase.
