# Supplementary material for: Red Anthocyanins and Yellow Carotenoids Form the Color of Orange-Flower Gentian (Gentiana lutea L. var. aurantiaca)
Source: PLoS One. 2016 Sep 2;11(9):e0162410. doi: 10.1371/journal.pone.0162410 (PMC5010251; doi:10.1371/journal.pone.0162410)
Supplement: S2 Table — Abbreviations: CHS, chalcone synthase; CHI, chalcone isomerase; F3H, flavonone 3-hydroxylase; DFR, dihydroflavonol 4-reductase; ANS, anthocyanidin synthase; 3GT, UDP-glucose:flavonoid-3-O-glucosyltransferase; F3'H, flavonoid 3'-hydroxlase; F3'5'H, flavonoid 3',5'-hydroxylase. (DOC) [file pone.0162410.s007.doc]

**S2 Table. Oligonucleotide sequences of primer pairs designed based on the cDNA sequences isolated from petals of *Gentiana lutea* L. var. *aurantiaca*, and ubiquitin (*UBQ*) gene [39] as reference used for quantitative real-time PCR (qRT-PCR) analysis.**

| Genes | Direction | Sequence (5´→ 3´) |
| --- | --- | --- |
| *CHS* | Forward | AAAAACTGCGGTCTACGAGGC |
|  | Reverse | CTTGCTCCATCTCTTGCTGAA |
| *CHI* | Forward | AAAGGTGGCGGAAAATTGC |
|  | Reverse | GCTCCATTCTTCCCAAAACT |
| *F3H* | Forward | GAAGCCGGTTCTCGATGAG |
|  | Reverse | TCTTTGCTCATCTTCCTCCG |
| *DFR* | Forward | GGGAATGAAGCACACTATGGT |
|  | Reverse | ATCTTCCTTCTGCTTCTGGGT |
| *ANS* | Forward | TACCCAAAGTGTCCTCAACCG |
|  | Reverse | TGCTGTGATCCATTTGTCTTG |
| *3GT* | Forward | GGGTAACAGTGATCGGTCGG |
|  | Reverse | TACTCCACCTTTCACCCCAAC |
| *F3´H* | Forward | TGGTGGAGAGAAGCCTAATGT |
|  | Reverse | AACGACTGAACTAACGAAGCC |
| *F3´5´H* | Forward | AAAGGAACTCGGCTCAACG |
|  | Reverse | ATCCCCATTCTTGTTCCAGC |
| *UBQ* | Forward | CGATTGATAATGTGAAAGCCAAAA |
|  | Reverse | TCCGGCAAATATCAACCTCTG |

Abbreviations: CHS, chalcone synthase; CHI, chalcone isomerase; F3H, flavonone 3-hydroxylase; DFR, dihydroflavonol 4-reductase; ANS, anthocyanidin synthase; 3GT, UDP-glucose:flavonoid-3-*O*-glucosyltransferase; F3'H, flavonoid 3'-hydroxlase; F3'5'H, flavonoid 3',5'-hydroxylase.
