# Supplementary material for: Red Anthocyanins and Yellow Carotenoids Form the Color of Orange-Flower Gentian (Gentiana lutea L. var. aurantiaca)
Source: PLoS One. 2016 Sep 2;11(9):e0162410. doi: 10.1371/journal.pone.0162410 (PMC5010251; doi:10.1371/journal.pone.0162410)
Supplement: S3 Table — Values are expressed as μg/g DW; Each value represents the mean result from three determinations from three different petal batches ±SD (standard deviation). (DOC) [file pone.0162410.s008.doc]

**S3 Table.** **Concentration of carotenoid composition and content in orange petals in *Gentiana lutea* L. var. *aurantiaca* during different stages of flower development**.

| Orange S1 S2 S3 S4 S5 |
| --- |
| Neox 6.39+0.17 7.57+1.62 8.21+0.15 9.05+0.75 5.81+0.35  Anth 22.43+0.59 20.30+4.36 26.00+0.46 30.20+2.84 19.58+1.18  Vio 5.62+0.41 5.33+1.16 8.21+0.19 10.24+0.80 10.29+0.62  Lut 87.80+2.29 84.11+12.09 74.22+3.305 101.62+7.55 70.22+4.23  Mono-a-car 2.94+0.08 2.58+0.54 3.59+0.11 5.48+0.45 4.81+0.29  b-Cry 4.32+0.12 6.31+1.22 7.35+0.23 10.95+0.45 9.30+0.56  b-Car 42.27+1.11 46.27+6.95 49.42+2.87 73.07+3.01 46.65+2.81 |
| **Total 171.77 172.47 177.00 240.66 166.66** |

Abbreviations: S, stage; Neox, neoxanthin; Anth, antheraxanthin; Vio, violaxanthin; Lut, lutein; Mono-a-car, monohydroxy α-carotenes; b-Cry, β-cryptoxanthin; b-Car, β-carotene. Values are expressed as µg/g DW; Each value represents the mean result from three determinations from three different petal batches +SD (standard deviation).
