# Supplementary material for: Red Anthocyanins and Yellow Carotenoids Form the Color of Orange-Flower Gentian (Gentiana lutea L. var. aurantiaca)
Source: PLoS One. 2016 Sep 2;11(9):e0162410. doi: 10.1371/journal.pone.0162410 (PMC5010251; doi:10.1371/journal.pone.0162410)
Supplement: S4 Table — Each value represents the mean result from three determinations from three different petal batches ±SD (standard deviation). (DOC) [file pone.0162410.s009.doc]

**Table S4** **Concentration of carotenoid composition and content in yellow petals of *Gentiana lutea* L. var. *lutea* during different stages of flower development**.

| Yellow S1 S2 S3 S4 S5 |
| --- |
| Neox 6.89+0.58 4.43+0.23 4.61+0.23 5.02+0.33 5.74+0.15  Anth 21.88+1.86 19.18+0.98 19.11+0.97 20.65+1.39 20.72+0.56  Vio 4.44*+*0.38 5.61+0.28 8.18+0.29 10.23+0.68 11.49+0.21  Lut 76.71+6.29 81.58+4.18 85.14+4.33 83.19+5.60 79.74+2.15  Mono-a-car 2.45+0.21 1.88+0.09 3.54+0.18 4.44+0.29 4.99+0.14  b-Cry 5.36+0.45 3.90+0.20 7.44+0.38 9.85+0.66 9.81+0.26  b-Car 35.95+3.06 41.18+2.11 49.21+2.51 59.73+4.13 52.54+1.42 |
| **Total 153.68 157.76 177.23 193.11 185.03** |

Abbreviations: S, stage; Neox, neoxanthin; Anth, antheraxanthin; Vio, violaxanthin; Lut, lutein; Mono-a-car, monohydroxy α-carotenes; b-Cry, β-cryptoxanthin; b-Car, β-carotene. Each value represents the mean result from three determinations from three different petal batches +SD (standard deviation).
